# Protocols for Metallo- and Serine-β-Lactamase Free Energy Predictions: Insights from Cross-Class Inhibitors

**DOI:** 10.1021/acs.jpcb.4c06379

**Published:** 2024-12-05

**Authors:** J. Jasmin Güven, Marko Hanževački, Papu Kalita, Adrian J. Mulholland, Antonia S. J. S. Mey

**Affiliations:** † EaStCHEM School of Chemistry, University of Edinburgh, Edinburgh EH9 3FJ, United Kingdom; ‡ Centre for Computational Chemistry, School of Chemistry, University of Bristol, Bristol BS8 1TS, United Kingdom

## Abstract

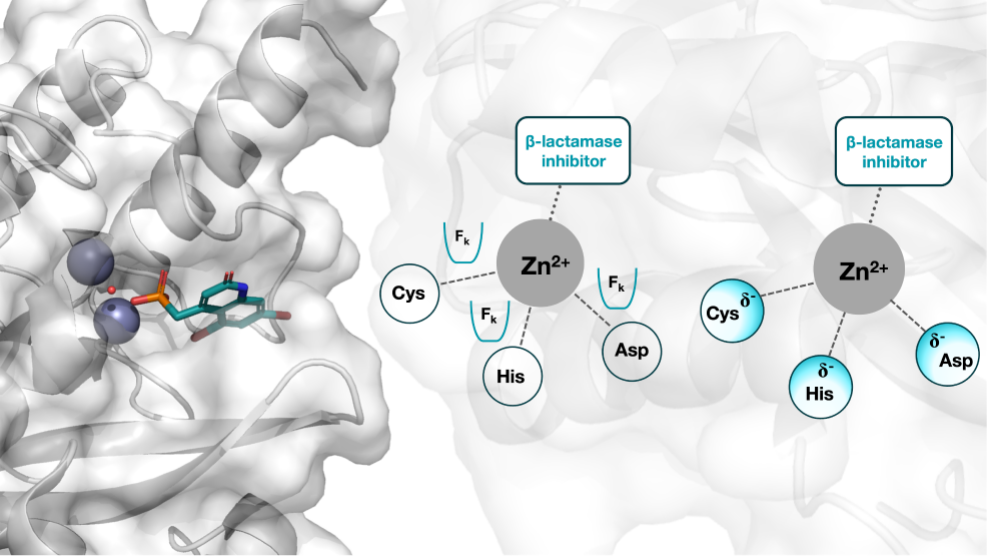

While relative binding free energy (RBFE) calculations using alchemical methods are routinely carried out for many pharmaceutically relevant protein targets, challenges remain. For example, open-source tools do not support the easy setup and simulation of metalloproteins, particularly when ligands directly coordinate to the metal site. Here, we evaluate the performance of RBFE methods for KPC-2, a serine-β-lactamase (SBL), and two nonbonded metal parameter setups for VIM-2, a metallo-β-lactamase (MBL) with two active site zinc ions. We tested two different ways of modeling the ligand–zinc interactions. First, a restraint-based approach, in which FF14SB zinc parameters are combined with harmonic restraints between the zincs and their coordinating residues. The second approach uses an upgraded Amber force field (UAFF) for zinc–metalloproteins with adjusted partial charges and nonbonded terms of zinc–coordinating residues. Molecular mechanics (MM) and quantum mechanics/molecular mechanics (QM/MM) simulations show that the crystallographically observed zinc coordination is not retained in MM simulations with either zinc parameter set for a series of known phosphonic acid–based inhibitors bound to VIM-2. These phosphonic acid–based inhibitors exhibit known cross-class affinity for SBLs and MBLs and serve as a benchmark for RBFE calculations for VIM-2, after validation with KPC-2. The KPC-2 free energy of binding estimates are within expected literature accuracies for the ligand series with a mean absolute error of  kcal/mol and a Pearson’s correlation coefficient of . For VIM-2, the UAFF approach has improved correlation from  to , compared to the restraint approach. The presented strategies for handling ligands coordinating to metal sites highlight that simple metal parameter models can provide some predictive free energy estimates for metalloprotein–ligand systems, but leave room for improvement in their ease of use, modeling of coordination sites and as a result, their accuracy.

## 1 Introduction

β-Lactam antibiotics, such as carbapenems and penicillin work by inhibiting bacterial penicillin-binding proteins, and are often used as “last-resort” antibiotics.^1^ As a result, bacteria that produce carbapenem hydrolyzing β-lactamases have become a growing global healthcare concern.^2^ To address this, the development of combination therapeutics has become a well-established method for extending antibiotic efficacy. For example, avibactam was approved in 2015 by the FDA as an inhibitor for TEM-1, a class A β-lactamase commonly found in resistant bacteria.^3,4^ However, while avibactam can be active against SBLs in classes A, C, and D, including KPC-2, it has no activity against MBLs. Furthermore, while an MBL inhibitor developed by Merck has reached Phase I trials,^5^ there are currently no clinically approved inhibitors against MBLs.^6^

Computational tools can aid in the design of new inhibitors, as well as predicting absorption, distribution, metabolism, and excretion (ADME) properties to speed up and reduce the cost of early stage drug discovery. Part of such a computational toolbox are alchemical perturbation methods. They provide an effective strategy for computing free energies of binding between proteins,^7^ or proteins and ligands.^8^ With both open source^9,10^ and commercial^11,12^ tools available, these methods have been widely adopted across both academia and industry.^13,14^

Particularly relative binding free energy (RBFE) methods can provide reliable and accurate results with high-rank correlations (*R* > 0.7) and low root-mean-square errors (RMSE) (<1 kcal/mol) in free energy calculations with respect to experiment for many protein systems.^15^ RBFE methods have seen wide adoption for free energy prediction in lead optimization, particularly in combination with active learning strategies, to drive down computational costs.^16^ However, some technical challenges, as well as system dependent ones remain. For example, despite the wide usage of soft-core potentials, handling singularities arising from appearing or disappearing particles remains an active area of research. As an alternative to soft-core potentials, neural network potentials may in the future be used to linearly interpolate between molecules without singularities from soft-core approaches; this, however, requires further research. Accounting for charge changes in transformations between two ligands^17−19^ also leaves room for improvement. Other issues stem from standard force fields used, for example, halide parameters often do not account for σ holes well.^20^ Another challenging area is metalloproteins with no established RBFE strategies for modeling ligands that coordinate to metals using open-source tools.

In this paper, we explore ways to model metalloproteins with ligand-coordinated metal sites using RBFE with open-source tools. Recent commercial force fields, such as OPLS-5^21^ discusses improved modeling capabilities for metals, but are not available open source. We tested two force field approaches for modeling coordinating metals in RBFE calculations for metalloproteins. While we look at divalent zinc proteins, the strategies proposed can be applied to other metalloproteins but will need further validation.

Since there is a need for pan-β-lactamase inhibitors, understanding what makes a good cross-class inhibitor and how to design for them is essential. One corner stone for this is being able to estimate how reliable binding affinity estimates are for already known inhibitors. We use a set of ligands with cross-protein class inhibition against serine- and metallo-β-lactamases, identified by Pemberton et al.^1^ It is comprised of heteroarylphosphonates with affinity for both a serine-β-lactamase (KPC-2) and two metallo-β-lactamases (NDM-1 and VIM-2). These ligands have a good dynamic range across the two enzyme classes and act as a good benchmark set for β-lactamases. For these ligands, we demonstrate that RBFE methods for serine-β-lactamases are accurate at predicting binding affinity, in line with other benchmarking.^15^ For VIM-2, we test a restraint-based method and an augmented parameter set for Amber-based zinc force fields using the same ligand force field parameters.

## 2 Theory

We are often interested in studying the effects of small structural changes on the binding affinity of a series of structurally similar drug-like molecules. Experimentally, this is often done in structure–activity relationship (SAR) studies, while computationally we can use molecular dynamics (MD) methods. Simulating binding events directly with unbiased MD requires the observation of multiple binding events for sufficient statistics and due to the time scales of MD, more efficient methods are needed.^14^ One class of such methods is relative binding free energy (RBFE) calculations. These make use of a thermodynamic cycle to evaluate a relative free energy of binding , by simulating an unphysical path of an *alchemical* transformation of one molecule into another molecule. By computing the free energy of this transformation in a water box (), and while *bound* to the protein (), a relative free energy of binding (), defined as a difference between the binding free energies  and , can be computed for a given ligand pair *L*_1_ and *L*_2_.
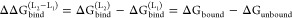
1Typically a parameter λ is used to control the alchemical transformation from a ligand *L*_1_ to a ligand *L*_2_ in these relative alchemical free energy methods. Briefly, the λ parameter controls the potential energy function so that at values λ_1_ and λ_2_, the potential for ligands *L*_1_ and *L*_2_ is recovered.^14^ More details on RBFE calculations and best practices can be found in Mey et al.^14^ and Hahn et al.^15^

### 2.1 Modeling Metals Can Be a Challenge for Alchemical Free Energy Calculations

Modeling metals is difficult not only with RBFE calculations but also with other MD methods. This is especially true for transition metals, where molecular mechanics (MM) force fields do not correctly describe the interactions between the metal and coordinating residues. Some existing metal parametrization options include bonded, nonbonded, and cationic dummy atom models.^22^ In the bonded model, the metal–ligand coordination bonds are modeled as explicit, covalent bonds, which makes them unsuitable for RBFE calculations involving ligand transformations,^23^ but could open up new avenues for RBFE calculations using protein transformations. Cationic dummy atom models place dummy atoms around the metal center to describe the charge distribution of metals more accurately.^22^ While accurate, these models are not easy to set up, and as such, are not an attractive option for automated RBFE workflows. Finally, nonbonded models do not model the coordination bond between metals and ligands explicitly. Instead, some models make use of harmonic distance restraints and/or adjusted electrostatic and Lennard-Jones parameters.^23−26^ The different metal modeling options are reviewed extensively by Li and Merz^22^ and different models have been benchmarked by Melse et al.^26^

## 3 Methods

In the following, we describe the setup and workflow of molecular dynamics (MD) simulations, quantum mechanics/molecular mechanics (QM/MM) calculations, and the relative binding free energy (RBFE) calculations for the serine-β-lactamase KPC-2 and metallo-β-lactamase VIM-2.

### 3.1 System Preparation

**Figure 1:**
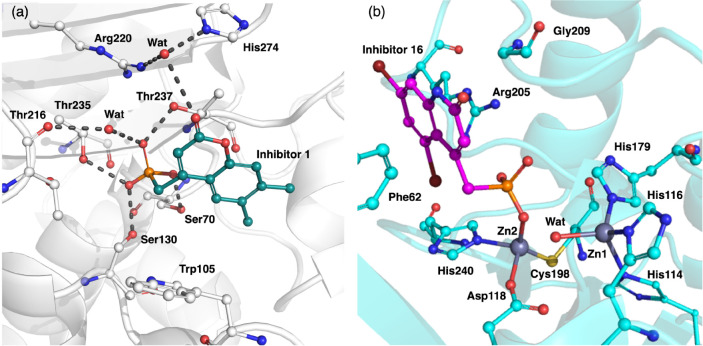
Active sites of the crystal structures of (a) KPC-2 (PDB ID 6D15), a serine-β-lactamase and (b) VIM-2 (PDB ID 6O5T), a metallo-β-lactamase. The structures also show the crystallized bound poses of (a) inhibitor 1 bound to KPC-2 and (b) inhibitor 16 bound to VIM-2. For KPC-2, the inhibitor forms hydrogen bonds (shown in dotted lines) with the catalytic Ser70 and other key residues. In VIM-2, the inhibitor directly coordinates to zinc 2.

For KPC-2 we chose PDB ID: 6D15 for its resolution (1.30 Å) and the higher PDB validation metrics, following best practices by Hahn et al.^15^Figure 1a shows the active site from the crystal structure of *ligand 1* bound to KPC-2, with hydrogen bonds suggested by Pemberton et al.^1^ shown in dotted lines. We used Flare by Cresset^12,27^ to load in the PDB structure and select the more populated alternate side chain locations, based on electron density maps visualized in Flare. We used pdb4amber from AmberTools 22^28^ to fix the atom numberings in the PDB file before protonating amino acid side chains with the H++ server.^29^ We retained all crystallographic waters and the disulfide bridge between Cys69 and Cys238 for the simulations. The crystallographic glycol molecule and additional copies of *ligand 1* were removed. The crystal structure of *ligand 1* bound to KPC-2 was used as the template pose from which the 15 other ligands studied in Pemberton et al.^1^ were generated using Flare. For the KPC-2 simulations, the phosphonate group was simulated as singly protonated, as suggested by Pemberton et al.^1^

Figure 1b shows the active site from the crystal structure of VIM-2, which is a B1 type metallo-β-lactamase and has two Zn^2+^ ions in its active site. Zinc 1 coordinates to three histidine residues, the H3 site, whereas zinc 2 coordinates to aspartate, cysteine, and histidine, known as the DCH site.^30^ For VIM-2 (PDB ID: 6O5T), we retained chain A and prepared the protein in the same way as KPC-2, using Flare. We used the bound crystal structure of *ligand 16* as a template for the other ligands, as this structure was crystallized using initial soaking conditions at a pH of 7.5. Following Pemberton et al.,^1^ the VIM-2 simulations were carried out with the phosphonate group of the ligand fully deprotonated. We ran QM/MM simulations (described below) to verify if the bridging water molecule, coordinating to one of the zinc ions, should be modeled as a water molecule or a hydroxide ion.

### 3.2 QM/MM Simulations for VIM-2 Active Site

To test the presence of a single water/hydroxide molecule and the protonation state of the phosphonate group of the ligand, we performed QM/MM simulations starting from the MM equilibrated structures using DFTB3 in Amber.^31,32^ The QM region was defined by the active site of VIM-2, i.e., the two zinc ions, the ligand, the single water molecule, and the zinc-coordinating residues up to their β-carbons. The covalent bonds at the QM/MM boundary were treated using the link atom approach, the default scheme in Amber22.^32^ In addition, we performed a QM/MM simulation by replacing the active site water with a hydroxide ion to study its stability. The total charge of the QM region was 0 and −1 for the water and hydroxide systems, respectively. The spin multiplicity was 1 in both cases. Each system was initially minimized for a total of 1000 steps, followed by a 10 ps NPT relaxation and 100 ps production at 300 K using Langevin dynamics, implemented in sander.MPI from Amber22.^32^ To evaluate the stability of the zinc coordination, we analyzed zinc coordination distances and performed visual inspections of the active sites.

### 3.3 Ligand Setup

After generating and aligning the ligand series to the crystal structure of both proteins with Flare,^12,27^ we parametrized each ligand using antechamber and parmchk2 from AmberTools 22^28^ with GAFF 2 atom types and AM1-BCC charges, keeping the ligand charges as −1 e and −2 e for KPC-2 and VIM-2, respectively.

The inhibition constants (*K*_i_) of each of the ligands against KPC-2 and VIM-2 are shown in Table S1 in the Supporting Information. Using the approximation ,^15^ the *K*_i_ values were converted to  values using
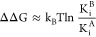
2from Hahn et al.,^15^ where *k*_*B*_ is the Boltzmann constant, *T* is the temperature and  and  are the inhibition constants of molecules A and B, respectively.

### 3.4 Zinc Parameters for VIM-2

We used two approaches to model the two zinc ions in the active site of VIM-2 to be able to evaluate their accuracy for RBFE calculations. The first approach is a restraint-based hybrid nonbonded model, where the zinc coordination bonds are replaced by harmonic restraints, as described by Li and Merz,^24^ while the rest of the protein is parametrized with the standard and well established Amber ff14SB force field. To avoid adding a bias to the RBFE predictions, the distance between the zinc and the ligand is not restrained.

The second approach is based on the upgraded Amber force field (UAFF) parameters for zinc-binding residues,^23,25^ which uses Amber force field ff14SB^33^ together with modified parameters for the zincs and their coordinating residues. UAFF models the polarization effects on zinc-coordinating residues with redistributed partial charges, and has been used to evaluate the binding free energy of a carboxypeptidase inhibitor.^25^ We combined the UAFF GROMACS compatible files from Macchiagodena et al.^23,25^ with the protein ff14SB force field and updated the missing parameters of the δ-protonated histidine dihedrals manually as suggested by Zheng.^34^ Similarly, the ffbonded.itp file given in Macchiagodena et al.^23,25^ for the GLZ and ASZ residues was edited to include the carbon atom (atom type CT) to which the zinc-coordinating oxygen (atom type OD1) is bonded. For editing the Gromacs ff14SB files, we followed the Gromacs instructions for adding a custom residue to a force field. Following the instructions in Macchiagodena et al.,^23,25^ the zinc-coordinating residues have been renamed to their corresponding UAFF names in our input pdb-file. After parametrization, we converted the Gromacs-formatted topology and coordinate files into Amber format with BioSimSpace.^9^ Both RBFE and MD simulations were carried out for both the restraint approach and the UAFF method.

### 3.5 Molecular Dynamics Simulations of KPC-2 and VIM-2

We ran 50 ns of MD with Amber22 to investigate the stability of the active site for KPC-2 and VIM-2 for all ligands and force field approaches. We have followed the workflow given in an Amber tutorial,^35^ with small changes to the number of equilibration stages. For the proteins, the Amber force field ff14SB was used, unless stated otherwise. All ligands were parametrized with GAFF 2 (see above). The KPC-2 systems were solvated using Gromacs, implemented in BioSimSpace 2024.1.(dev),^9^ and the VIM-2 systems were solvated using tleap from AmberTools 22.^28^ TIP3P water and Na^+^ counterions were used for both solvations.

For each system, we first used steepest descent to minimize the water for 5000 steps, followed by a 1 ns heating of the system to 300 K in the NVT ensemble using a Langevin thermostat and a time step of 1 fs. This was followed by an NPT equilibration with the Berendsen barostat and a time step of 1 fs, to allow the box density to relax. The nonbonded cutoff was 8 Å and the long-range interactions were calculated with Particle-mesh Ewald. We then reduced the position restraints of the solute from 100 kcal mol^–1^ Å^–1^ to 10 kcal mol^–1^ Å^–1^. The 10 kcal mol^–1^ Å^–1^ backbone restraints were then relaxed for 1 ns before reducing them to 1 kcal mol^–1^ Å^–1^ and to 0.1 kcal mol^–1^ Å^–1^ over 1 ns, each. Finally, the system was relaxed without positional restraints for 1 ns before the final 50 ns production run, using a 2 fs time step.

### 3.6 RBFE for KPC-2 and VIM-2

For RBFE calculations of KPC-2, and VIM-2 using the restraint approach, we followed the standard workflows given in Mey et al.^14,36^ and Hahn et al.^15^ to test how well standard RBFE methods and force field parameters perform for metalloenzymes. The perturbation network (see the Figure S15) was generated using LOMAP^37^ v.3.1. For VIM-2 RBFE simulations of both zinc models, we have added additional edges (shown in teal in Figure S15) which allowed us to fully connect the network, as some of the ligands were not tested experimentally against VIM-2. Before the RBFE production runs, we minimized both the *bound* and *unbound* stages for 5000 steps. The water for both stages is equilibrated for 10 ps within the NVT ensemble using the Langevin thermostat and a time step of 1 fs. In the *bound* stage, we perform an additional backbone-restrained NVT equilibration for 25 ps, with a 2 fs time step before running an unrestrained NVT equilibration for 25 ps, again with a 2 fs time step, which is also carried out in the *unbound* stage. The NVT equilibration is followed by restrained and unrestrained NPT equilibrations for 200 ps, using the Berendsen barostat and a time step of 2 fs. For each transformation, we used SOMD from Sire^38^ for RBFE simulations and MBAR from alchemlyb^39^ in BioSimSpace^36^ as the free energy estimator. Each λ-window was run for 4 ns and we simulated 11 and 16 windows for KPC-2 and VIM-2, respectively, as these provided adequate overlap between λ-windows. Examples of overlap matrices for two KPC-2 simulations are shown in Figures S17 and S18, S20 and S21 and S23 and S24 for the restrained and upgraded models, respectively. The RBFE calculations were run in triplicate and all results shown in Section 4.4 show the average across the three runs with error bars showing the standard deviation across the runs. We use maximum likelihood estimation from cinnabar 0.4.1^40^ to convert relative binding free energies to binding free energies.

For the UAFF approach, the RBFE equilibration protocol was modified slightly to keep the zinc coordination site better conserved. The unrestrained NVT equilibration run was removed entirely and additional positional restraints based on the zinc-residue distances from the crystal structure were added to the two zincs, their coordinating protein residues, and the ligand, to ensure the zinc coordination did not change drastically before starting the RBFE production runs. All of the code to carry out simulation setups, excluding the QM/MM simulations, is available on GitHub at https://github.com/meyresearch/metalloenzymes/tree/main. Examples of input files for all the simulations, including QM/MM, are available on GitHub at https://github.com/meyresearch/alchemistry_with_betalactamases/tree/main.

## 4 Results and Discussion

In the following section, we show the results from MD simulations, QM/MM calculations, and the RBFE calculations for KPC-2, and VIM-2 using the two zinc modeling options.

### 4.1 KPC-2 MD Simulations Show Stable Complexes and RBFE Calculations Give Accurate and Well-Correlated Results

We performed 50 ns MD simulations of all protein–ligand complexes of KPC-2 to evaluate the stability of protein–ligand interactions. The *ligand 1* root-mean-square deviation (RMSD) (see the Figure S7) shows that the ligand stays bound to KPC-2 throughout the 50 ns MD simulation. Some of the earlier fluctuations of the RMSD are explained by the fact that the simulation starts with the ligand having π–π interactions with Trp105, which fluctuates for the first 10–15 ns. After this, the ligand settles into a π–π interaction with Trp105 for the duration of the MD simulation. Other ligand RMSDs are shown in Figure S7. Generally, all ligands remain stably bound to the protein.

**Figure 2:**
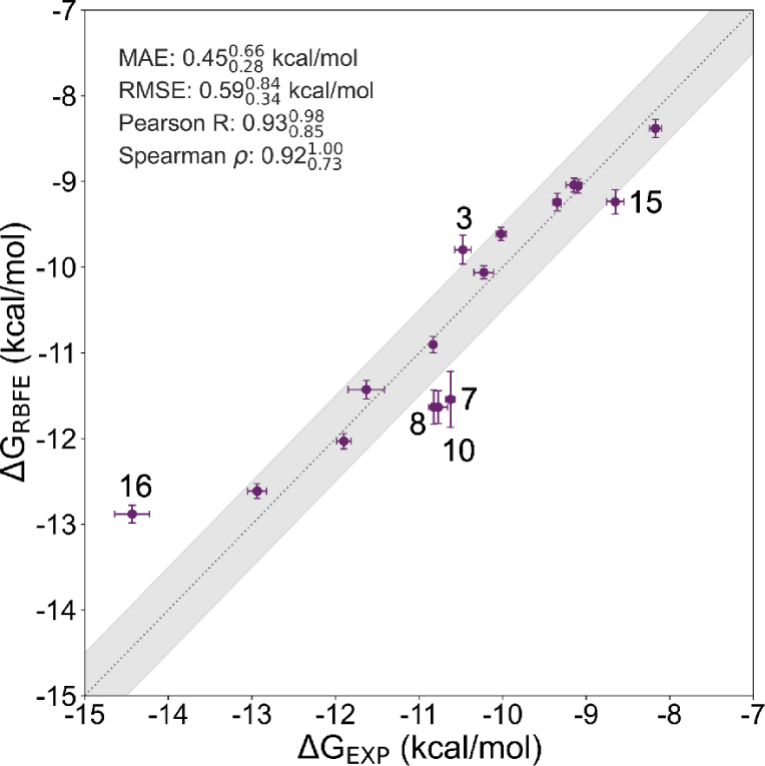
Correlation between experimental Δ*G*s and computed Δ*G*s as described above. The shaded region shows the 1 kcal/mol error lines. Outliers are identified by their ligand number as shown in Table S1.

Figure 2 shows the correlation between experimental free energies of binding and calculated relative binding free energies converted to free energies of binding. Figure S16 in the shows the calculated RBFE (averaged over the three repeats) against experimental RBFE (converted from *K*_i_ using eq 2). The error bars show the standard deviation from the three repeats. For the resulting binding free energy in Figure 2 the mean absolute error (MAE) of  kcal/mol is well below typically expected accuracies of 1 kcal/mol. Furthermore, Spearman’s rank correlation () and Pearson’s correlation coefficient (R) show that the RBFE calculations, obtained with existing ligand and protein force fields can predict experimental results well. In addition, the overlap matrices (see Figures S17 and S18) show sufficient overlap between consecutive λ-windows, suggesting that the free energy estimates obtained with MBAR are reliable.^14^

The outliers in Figure 2 and in the Figure S16, all (except *ligand 3* and *ligand 15*) involve transformations with halides, such as fluorine and bromine, which are known to be a challenge for small-molecule force fields, such as GAFF2.^20^ Additionally, transformations involving *ligand 3* involve a ring formation or breaking, which are known to be difficult transformations.^14^ Overall we standard RBFE calculations with the chosen force fields perform well for serine-β-lactamases.

### 4.2 QM/MM Validates Coordination and Active Site Modeling Choices

**Figure 3:**
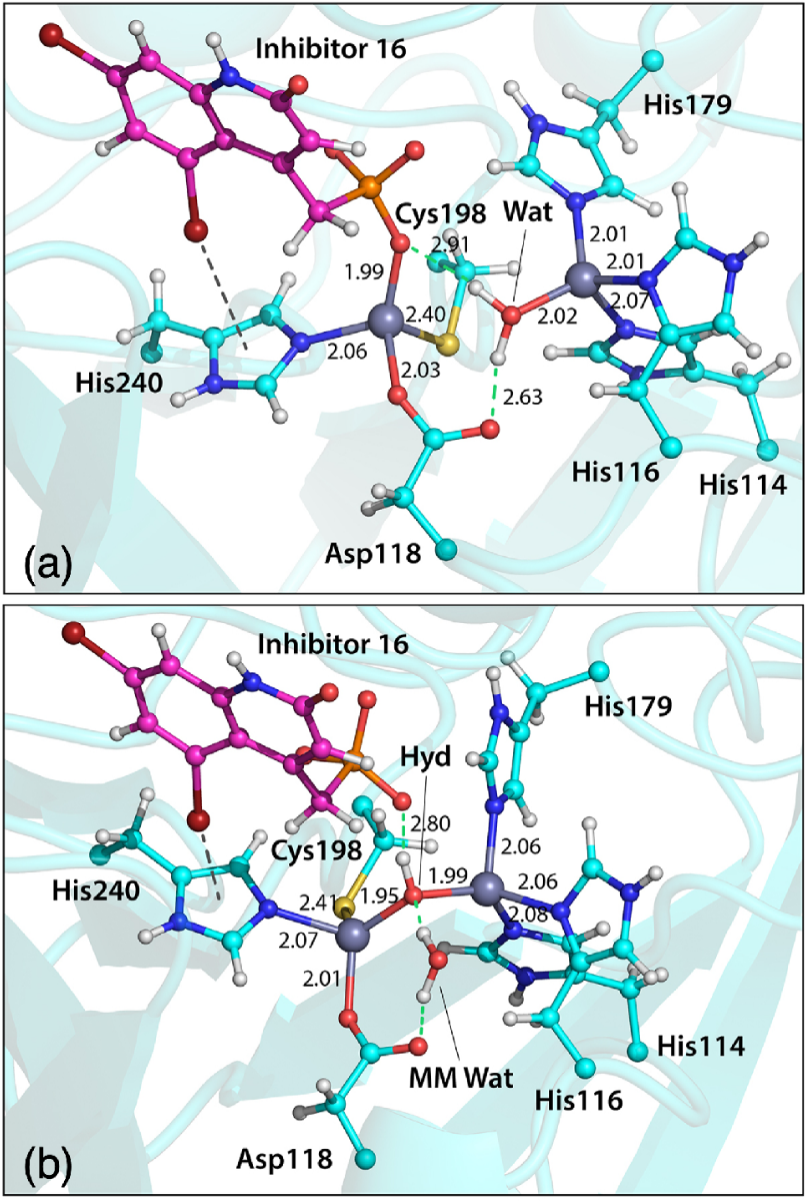
A representative snapshot of the VIM-2 active site with *ligand 16* bound obtained after 100 ps QM/MM simulations with (a) a water molecule and (b) a hydroxide ion. This confirms that a water molecule is more likely to be found in the active site of VIM-2 in the presence of the inhibitor due to QM/MM simulations reproducing direct coordination of *ligand 16* to the DCH zinc site as observed in the crystal structure. The alternative scenario with a bridging hydroxide caused significant deviations of the active site from the crystal structure and breaking of direct coordination with the phosphonate group of the inhibitor.

The stability of the binding site in the presence of water and hydroxide was investigated with QM/MM MD simulations and compared to the starting crystal structure of VIM-2 with *ligand 16* (see Figure 1b). While it is clear that the phosphonate group of the inhibitor should be fully deprotonated when found near two positively charged zinc ions at neutral pH, the protonation state of the catalytic water is less obvious. Our simulations suggested that there should be one water molecule near the phosphate group which was confirmed by obtaining a stable geometry of the active site during 100 ps of QM/MM simulation that closely resembled the crystal structure (Figure 3a).

On the other hand, the presence of the hydroxide ion significantly disrupted the geometry of the active site, especially the inhibitor coordination with the zinc, due to a strong preference for the hydroxide for bridging the two zinc ions and the electrostatic repulsion with the negatively charged phosphate group as shown in Figure 3b and in the Figure S4. Interestingly, the active site with the hydroxide was not only different from the starting crystal structure but also experienced more flexibility as two distinct conformations were characterized with the RMSD and distance analysis (see the Figure S5.). The zinc–zinc distance in the water simulations remains at (4.7 ± 0.2) Å, close to the distance of 4.45 Å from the crystal structure. In the hydroxide simulations, this distance is (3.6 ± 0.1) Å, which is significantly different from the crystal structure distance.

The main stabilizing interactions between the inhibitor and the protein include hydrogen bonds of the phosphonate group with Arg120 and Asn210, and stacking with the aromatic Phe62 and Tyr67 residues. The bromine substitution in the R_3_ position remained in close proximity to the His240 indicating a stabilizing interaction between the σ-hole of the halogen and the electron-rich π-cloud of the histidine. These findings are also in line with Pemberton et al.^1^ As a result a bridging water molecule and not hydroxide ion will be used in all subsequent MM calculations.

### 4.3 VIM-2 MD Simulations Show How the UAFF Approach Improves on the Shortcomings of the Restraint Method

**Figure 4:**
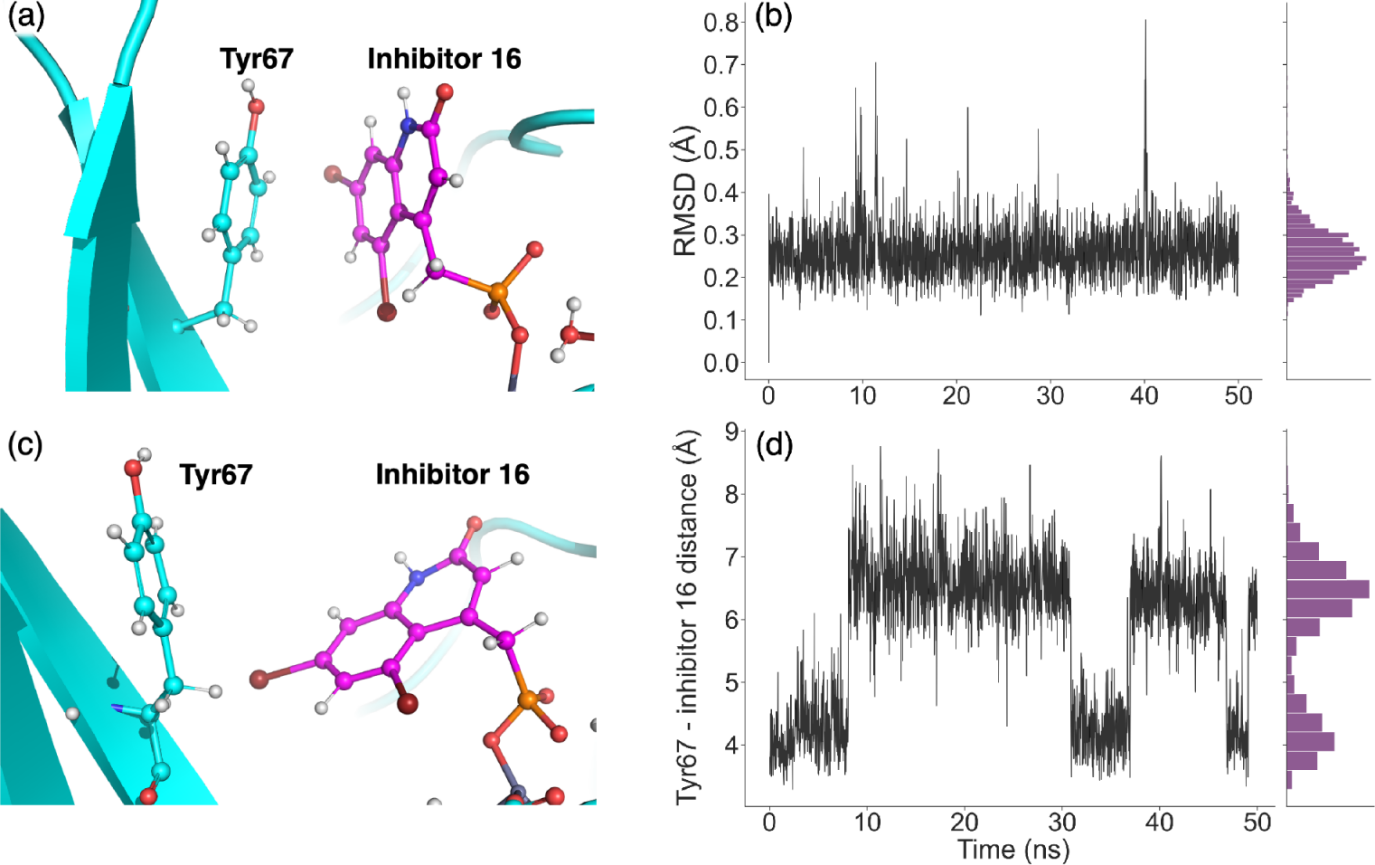
(a) *Ligand 16* pose with respect to the Tyr67 residue, indicating π–π-interaction with a dashed line. (b) RMSD of *ligand 16* bound to VIM-2 obtained from 50 ns standard MD simulations with the restraint approach. (c) Snapshot of the MD trajectory at 10 ns showing a change in both the ligand pose and Tyr67 pose. (d) Distance between the Y67 residue and *ligand 16* during the 50 ns MD simulation with the restraint approach.

Figure 4a shows the crystal structure of *ligand 16* bound to VIM-2, with the suggested π–π interaction with the Tyr67 residue also shown. In Figure 4b we show the RMSD of *ligand 16* bound to VIM-2 simulated for 50 ns with the restraint approach. Other ligand RMSDs are shown in the Figures S9 and S13 for the restraint, and UAFF approaches, respectively.

In Figure 4b, the RMSD is mostly stable with an average of (0.26 ± 0.06) Å. There are a few larger fluctuations around 10 and 40 ns. These fluctuations can be explained by a movement of the Tyr67 residue (see Figure 4c). At the start of the simulation, the face of the Tyr67 interacts with the face of the benzene ring of the ligand (see Figure 4a). However, at around 10 ns, Tyr67 swings away from the ligand, which allows the R_1_ bromine to take up this space, causing the ligand to swing toward Tyr67. Similarly, at around 37 ns, Tyr67 moves further away from the ligand, which then moves slightly closer to Tyr67, potentially to keep the π–π interactions. This movement can also be seen in the Tyr67-*ligand 16* distance shown in Figure 4d, obtained from the 50 ns MM MD simulation. Pemberton et al.^1^ also list Tyr67 as a key residue for ligand 16 binding to VIM-2. Furthermore, the R_3_–His263 σ–π stacking interaction present in the QM/MM and also mentioned by Pemberton et al.,^1^ is not present in the MD simulation. This could be explained by the fact that halides, such as bromine, are not well described by our chosen small molecule force field (GAFF 2) due to their σ holes.^20^

In contrast, the UAFF MD simulation shows a very stable *ligand 16* RMSD (see the Figure S12). The DCH site stays tetrahedral with the ligand sitting bidentate on the zinc. The H3 zinc site becomes octahedral with an additional water molecule and a nearby serine residue that coordinates to this zinc. These coordination changes are likely due to the fact that in the UAFF method, the zinc ion is still modeled with a point charge of +2 e, which may be too strong to keep the tetrahedral coordination from crystal structures, especially in the vicinity of neutral histidines.

### 4.4 Out-of-the-Box Restraint-Based Methods Have Some Predictiveness for Binding Affinities for VIM-2

**Figure 5:**
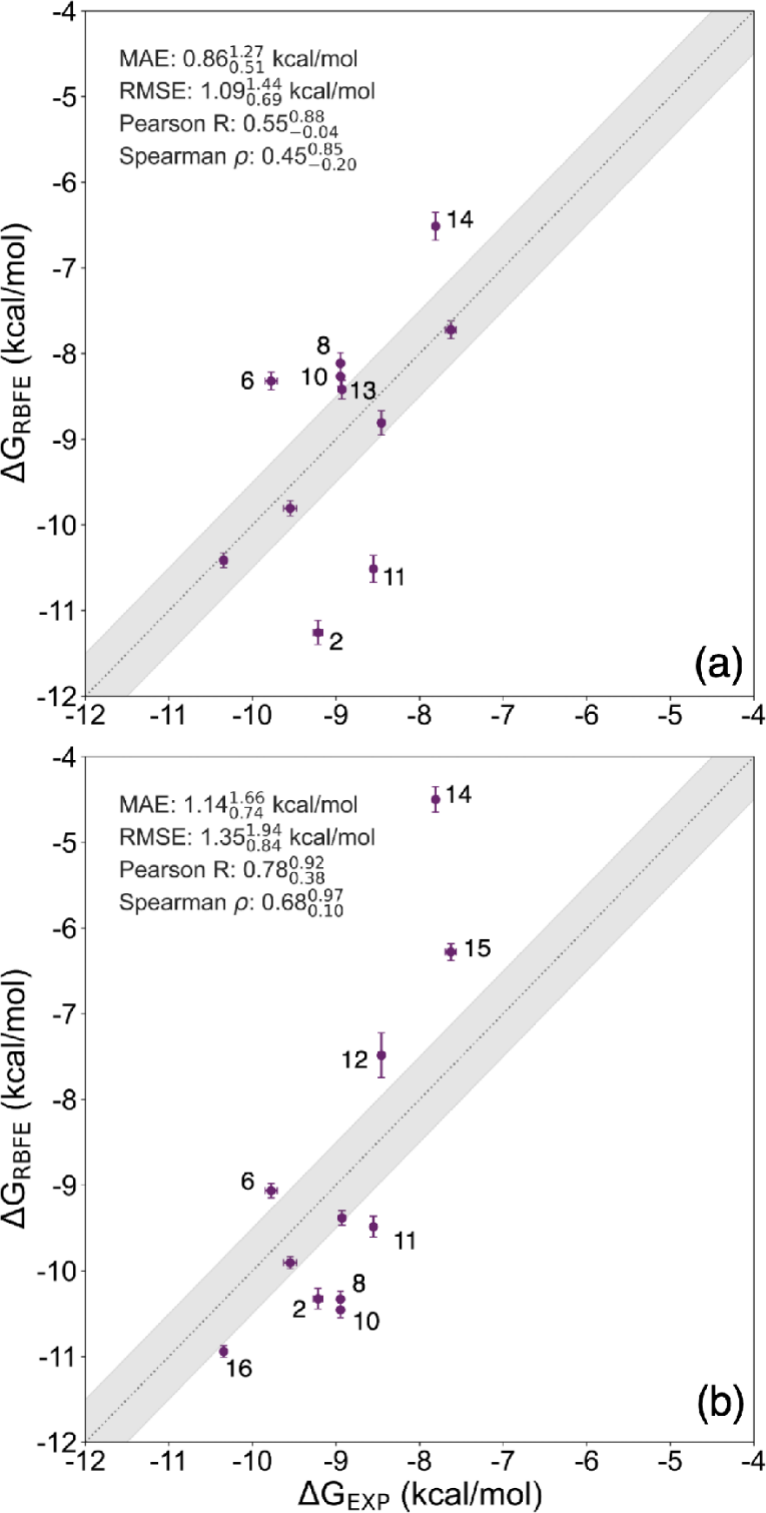
Correlation between experimental Δ*G*s and computed Δ*G*s, calculated with (a) the restraint approach and (b) UAFF for VIM-2. The shaded region shows the 1 kcal/mol error. Outliers are labeled according to their ligand number.

**Figure 6:**
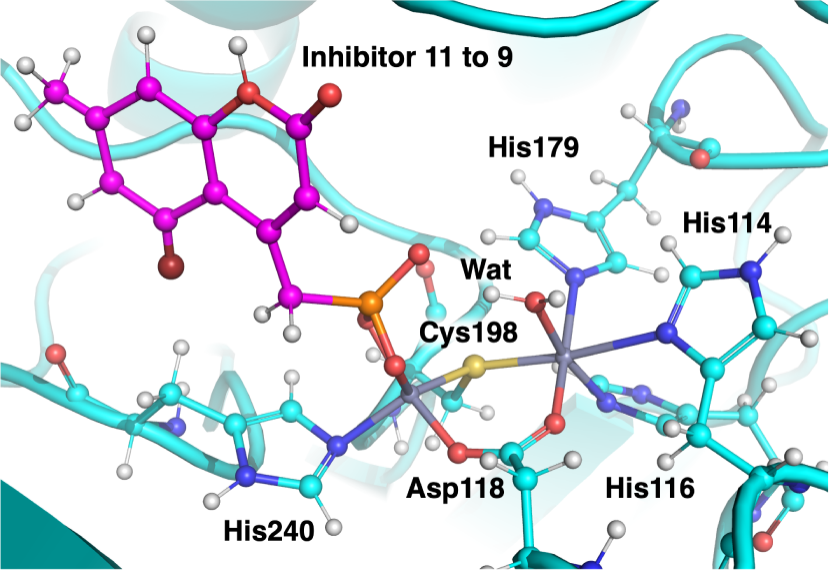
Snapshot at the end of the first λ-window for the transformation from *ligand 11* to *ligand 9*, which shows the coordination change occurring in most of the restraint model simulations.

Figure 5a shows the correlation between the experimental binding free energies and the calculated relative binding free energies converted to binding free energies obtained from simulations of VIM-2 with the restraint approach. Here, the correlation statistics show that the model is only somewhat predictive. Some of the outliers arise from similar issues as with KPC-2, such as ring breaking/formation and halides, but upon visually inspecting the trajectories, we see that the zinc coordination is not conserved during the RBFE simulations. With most of these simulations, the cysteine and aspartate from the DCH zinc site move between the two zincs, causing the H3 zinc site to become octahedral with three histidines, crystal water, cysteine, and aspartic acid. The ligand becomes bidentately coordinated to zinc and the coordination changes from tetrahedral to square pyramidal. Figure 6 shows an example of such a coordination change for the transformation between *ligand 11* and *ligand 9* at the end of the first λ-window. Furthermore, Figure S19 in the shows the calculated RBFE against experimental values.

Comparing the coordination geometries of the outlier transformations to those lying within the gray region in Figure 5a, we see that in the results that correlate better with experiments (i.e., within the gray region), the zinc coordination is much more conserved. In these simulations, the H3 site still becomes octahedral, with two additional water molecules coordinating to the zinc, but the DCH site remains tetrahedral with the ligand sitting bidentate to zinc. This suggests that the RBFE prediction is less sensitive to the changes in the H3 site as long as the DCH site remains tetrahedral. Furthermore, coordination changes are not unexpected with the restraint approach, as it does not capture the polarization effects of metal coordination.

### 4.5 Using UAFF Improves the Predictiveness of Free Energies of Binding

Figure 5b shows the correlation between the experiment and binding free energies obtained from alchemical free energy calculations with the UAFF method. Compared to the restraint approach (Figure 5a), there is an improvement in both Pearson’s correlation coefficient (R) and Spearman’s ρ. However, both the MAE and RMSE are higher than in the restraint approach. Figures S25-S28 show the comparison of bootstrap distributions for each statistic calculated for the UAFF and restraint models. As these distributions are skewed (non-normal), we carried out the Mann–Whitney U statistic between the UAFF and restraint models to determine if the differences in these statistics are statistically significant. Table S2 in the shows the Mann–Whitney U test statistic and its corresponding p-value for each statistic. As all p-values are below the chosen significance level of α = 0.05, we reject the null hypothesis that the two distributions are equal and state that the differences are statistically significant.

In the UAFF simulations, the DCH site remains much closer to the crystal structure than when compared to the restraint approach. This is likely due to not only the more accurate partial charges of zinc-coordinating residues but also due to the more restrained equilibration protocol. In all transformations, apart from 16 to 9 and 16 to 15, the DCH site is tetrahedral (as in the crystal structure) with a bidentate ligand. For the two transformations starting from *ligand 16*, we notice an extra water molecule coordinating with the DCH zinc. As found in the restraint approach, the H3 site is mostly octahedral, while sometimes becoming square pyramidal, as was also found with the MD simulations. These issues could again be due to modeling the zinc with a point charge of +2 e. Furthermore, similar to KPC-2 and the restraint approach, all of the ligands labeled in Figure 5b, apart from *ligand 12*, are involved in transformations containing halides.

Overall, the UAFF RBFE and MD results suggest that both the improved partial charges on the zinc-coordinating residues, as well as the more restrained equilibration improve the Δ*G* predictions. However, improvements are still needed, especially in accounting for the change in the ligand charge in the presence of the metal. Similarly, the zinc coordination sites may be better conserved with a model that also modifies the zinc charge to account for polarization effects more accurately.

## 5 Conclusions and Outlook

Different modeling strategies for MBLs, such as molecular docking^41^ to screen small molecule libraries and QM/MM simulations to refine MBL models^42^ have been reported in the past. Here we report, for the first time, results for MBL RBFE calculations using open-source software tools. Being able to routinely use RBFE calculations for MBLs specifically but metalloproteins in general will provide an essential tool for drug targeting of these class of proteins.

This paper takes the first step to making RBFE calculations for metalloproteins more routine by looking at a series of ligands with cross-class affinity to two classes of β-lactamases. We have shown that current, out-of-the-box methods and force fields give reliable RBFE results for a serine-β-lactamase (KPC-2), with excellent correlation to experiment (*R* = 0.93). We have also tested two nonbonded metal parametrization options and show, for the first time, results of RBFE calculations on metallo-β-lactamases. These results suggest that carrying out a more restrained equilibration, as well as accounting for polarization effects in zinc-coordinating residues gives more predictive (*R* = 0.78, upgraded Amber force field) results than when using ff14SB together with harmonic restraints (*R* = 0.55). Furthermore, through our extensive QM/MM, MD, and RBFE simulations, we highlight some of the main issues with parametrizing metalloproteins. For example, accounting for the polarization effects on the ligand, and on the metal ions themselves may further improve the accuracy of these simulations. In addition, applying angle restraints as well as distance restraints in the restraint model may help to keep the metal coordination conserved during simulations and makes this approach broadly applicable to metalloprotein systems. The UAFF approach should be transferable to other zinc-based metalloproteins since their reparametrization covers 96% of zinc proteins in the Protein Data Bank.^25^ However, different strategies would be needed to transfer this to other metalloproteins. In future work, to effectively design inhibitors across classes of β-lactamases understanding their mode of action in the different classes will be needed for targeted design choices. Having reliable computational methods for predicting binding free energies available will be essential for this goal. Different strategies can be explored in the future for improving the ease of use and reliability of alchemical free energy methods for MBLs in particular and metalloproteins in general. Considering QM/MM alchemical approaches will likely alleviate the coordination site issues, but will increase computational costs.^43^ Improving MM-based polarization and charge distribution may be an alternative route to approach this, or using machine learned potentials to replace the QM layer for the RBFE calculations.^44^ Ultimately any of the modeling advances will have to be made available in open source tools that streamline the setup, parametrization and calculations of alchemical free energy methods for pharmaceutically relevant metalloproteins, beyond zinc-based metalloproteins.

## Supporting Information Available

Additional plots for QM/MM, MD, and RBFE simulations (PDF)
