# Supplementary material for: Protocols for Metallo- and Serine-β-Lactamase Free Energy Predictions: Insights from Cross-Class Inhibitors
Source: J Phys Chem B. 2024 Dec 5;128(50):12416–24. doi: 10.1021/acs.jpcb.4c06379 (PMC11664573; doi:10.1021/acs.jpcb.4c06379)
Supplement: Supplementary file 1 — jp4c06379_si_001.pdf [file jp4c06379_si_001.pdf]

Supporting Information
Protocols for Metallo- and Serine-β-lactamase Free Energy Predictions: Insights from Cross-Class Inhibitors

J. Jasmin Güven,† Marko Hanževački,‡ Papu Kalita,‡ Adrian J. Mulholland,‡ and Antonia S.J.S. Mey†

†*EaStCHEM School of Chemistry, University of Edinburgh, EH9 3FJ, Edinburgh, United Kingdom*

‡*Centre for Computational Chemistry, School of Chemistry, University of Bristol, BS8 1TS, Bristol, United Kingdom*

E-mail:

Experimental K_i values for KPC-2 and VIM-2

Table S1: Inhibition constants for KPC-2 and VIM-2 obtained from Pemberton et. al.[1]

Ligand	KPC-2<br>K_i (μM)	VIM-2<br>K_i (μM)
1	32.9 ± 3.3	2.1 ± 0.1
2	8.4 ± 0.4	2.1 ± 0.2
3	15.3 ± 2.5	Not tested
4	23.3 ± 4.6	Not tested
5	154.4	Not tested
6	1.4 ± 0.2	0.82 ± 0.1
7	11.9 ± 1.1	Not tested
8	9.3 ± 1.8	3.3 ± 0.1
9	0.246 ± 0.047	1.2 ± 0.16
10	8.5 ± 0.3	3.3 ± 0.03
11	142.3 ± 22.3	6.4 ± 0.04
12	101.5 ± 8.6	7.5 ± 0.02
13	2.2 ± 0.8	3.4 ± 0.03
14	730.4 ± 87.4	22.3 ± 1.3
15	326.1 ± 56.8	30.3 ± 3.2
16	0.020 ± 0.007	0.316 ± 0.015

QM/MM simulation analysis

Water simulation

QM/MM simulations modelling the bridging water as a water in the active site.

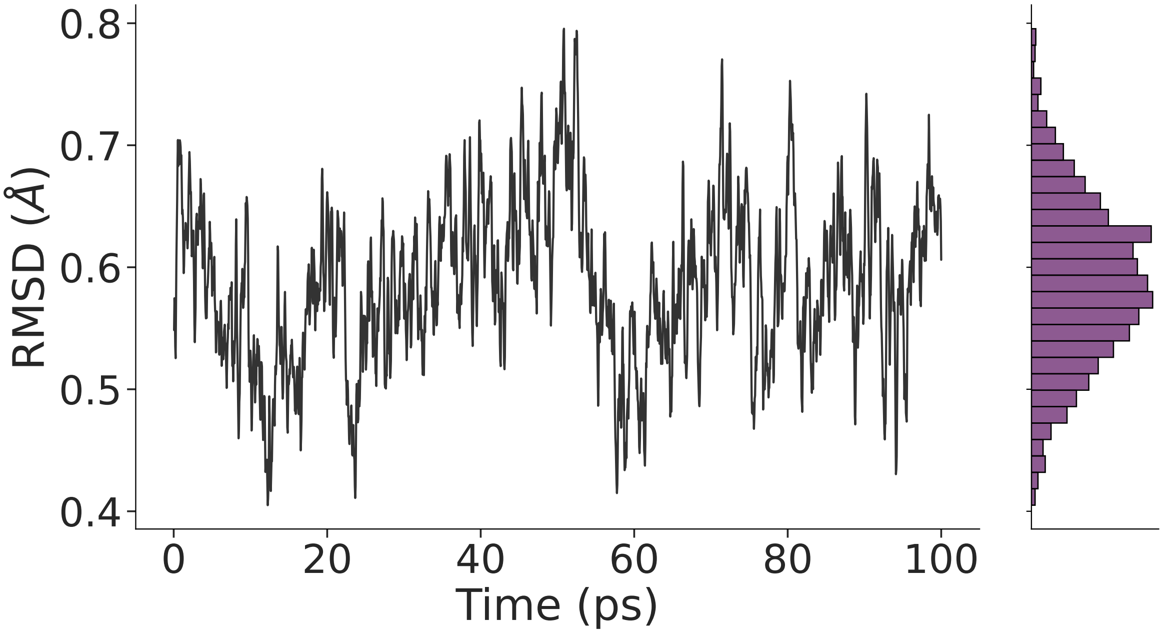

Figure S1: Active site RMSD from 100 ps QM/MM simulation of VIM-2 with *ligand 16* bound and with active site water molecule.

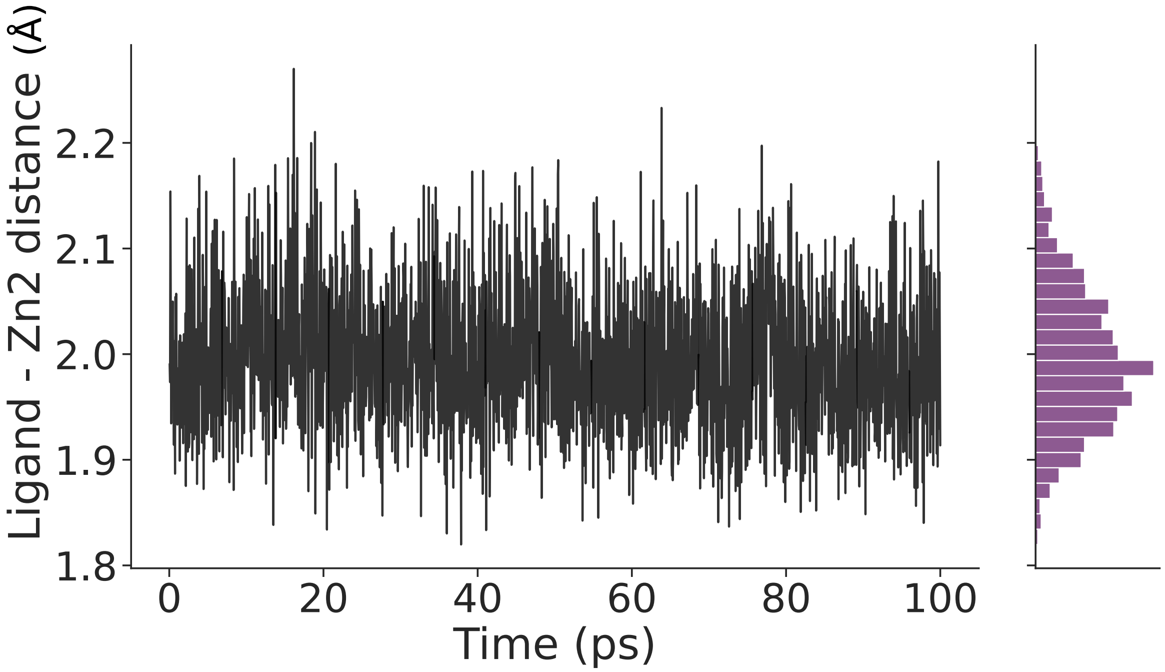

Figure S2: *Ligand 16* distance to zinc from 100 ps QM/MM simulation of VIM-2 with *ligand 16* bound and with active site water molecule.

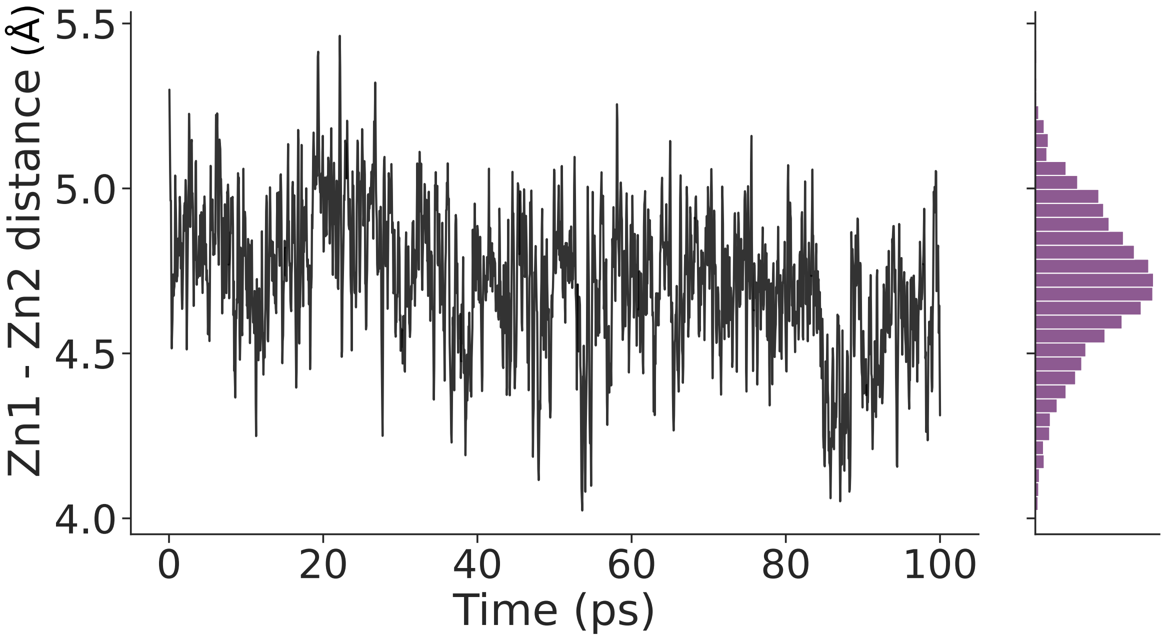

Figure S3: Zinc–zinc distance during 100 ps QM/MM simulation of VIM-2 with *ligand 16* bound and with active site water molecule.

Hydroxide simulation

QM/MM simulations modelling the bridging water as a hydroxie ion in the active site.

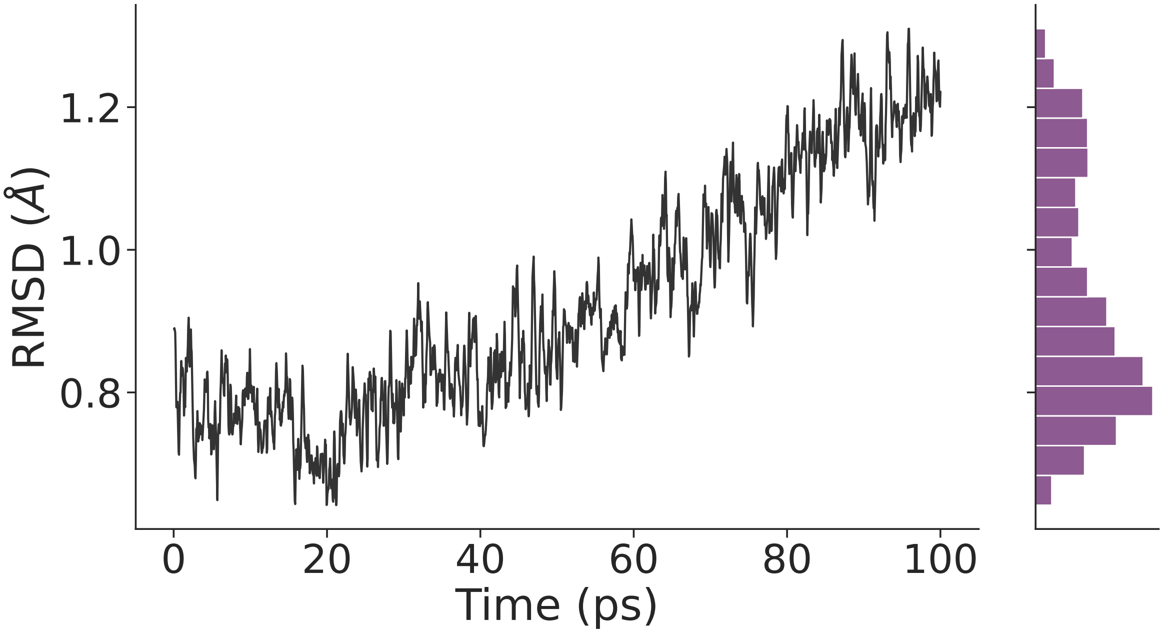

Figure S4: Active site RMSD during 100 ps QM/MM simulation of VIM-2 with *ligand 16* bound and with active site hydroxie ion.

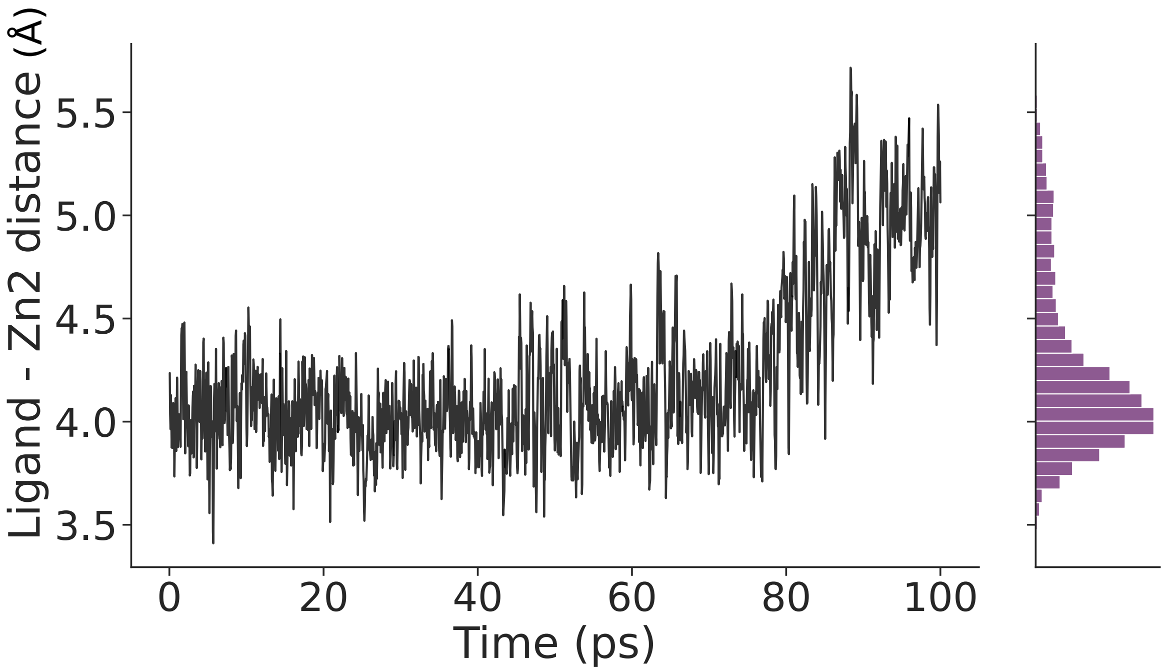

Figure S5: *Ligand 16* distance to zinc during 100 ps QM/MM simulation of VIM-2 with *ligand 16* bound and with active site hydroxide ion.

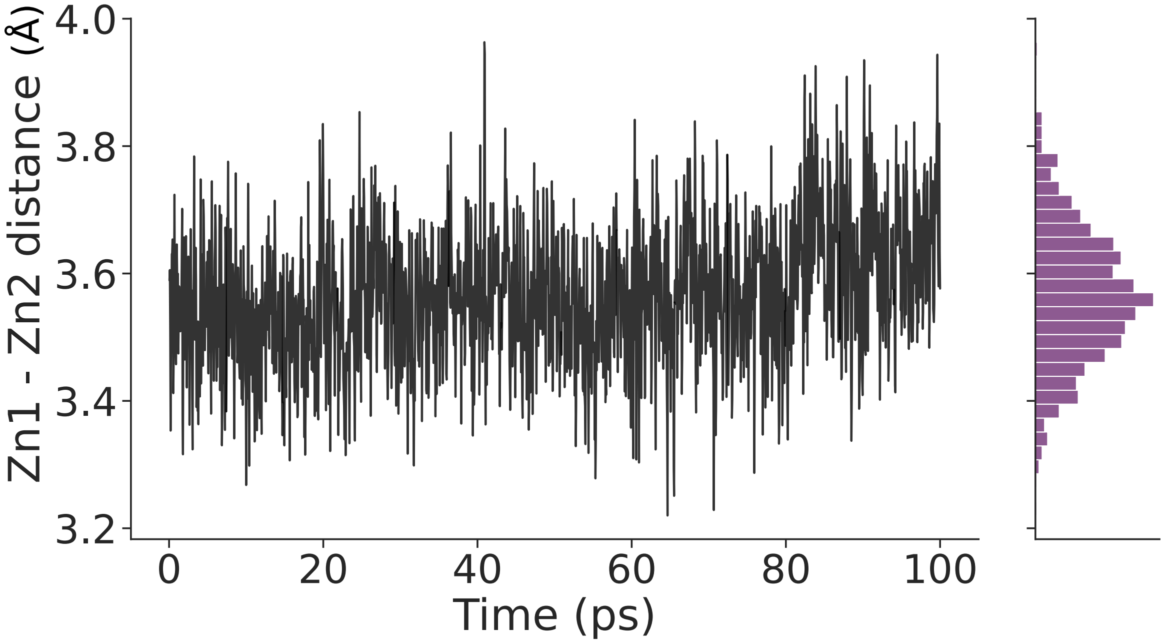

Figure S6: Zinc–zinc distance during 100 ps QM/MM simulation of VIM-2 with *ligand 16* bound and with active site hydroxide ion.

MD plots

Summary analysis of MD simulations of all ligands.

KPC-2

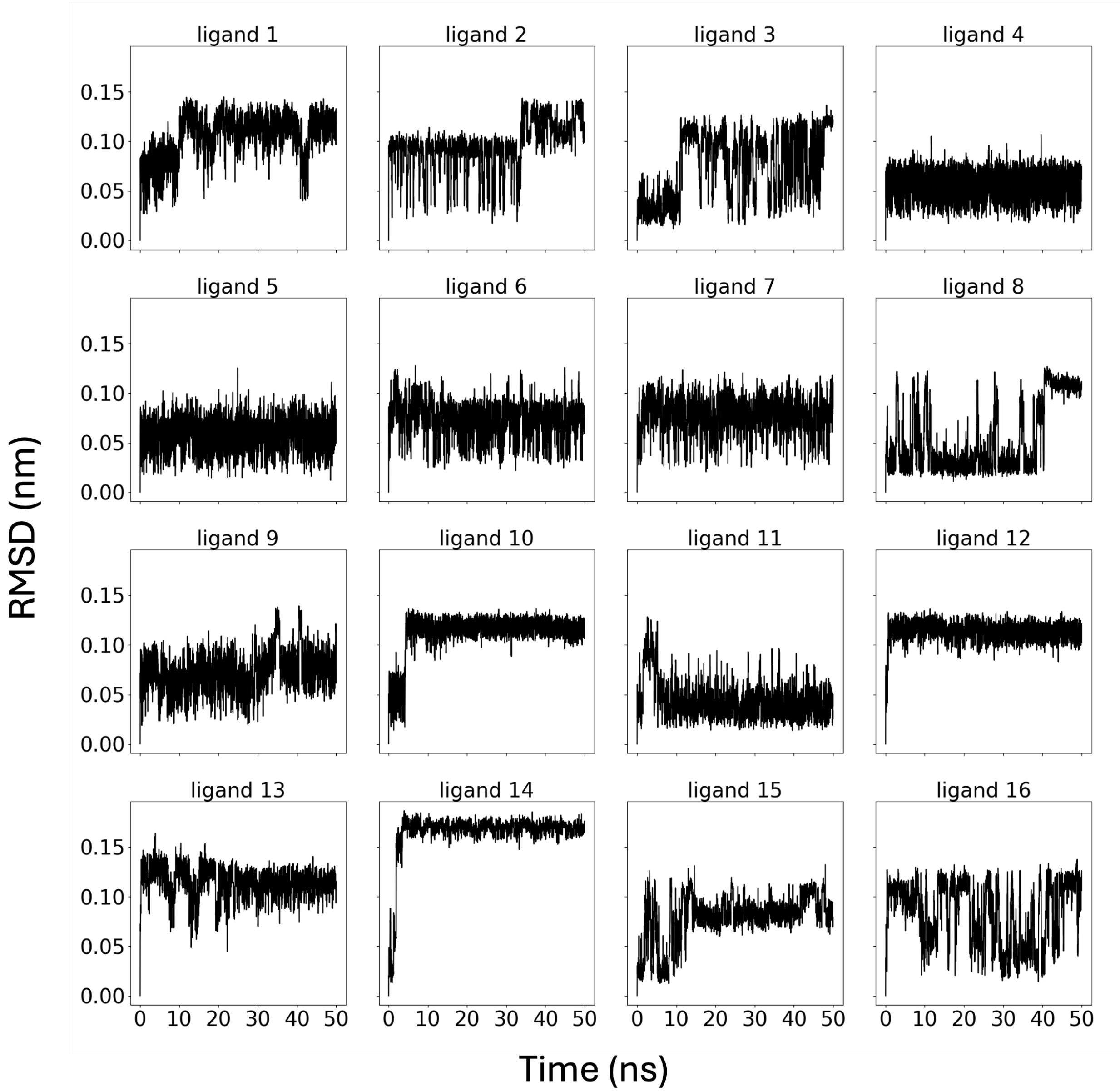

Figure S7: RMSDs computed from 50 ns MD simulations for each ligand bound to KPC-2.

VIM-2

The restraint-based approach

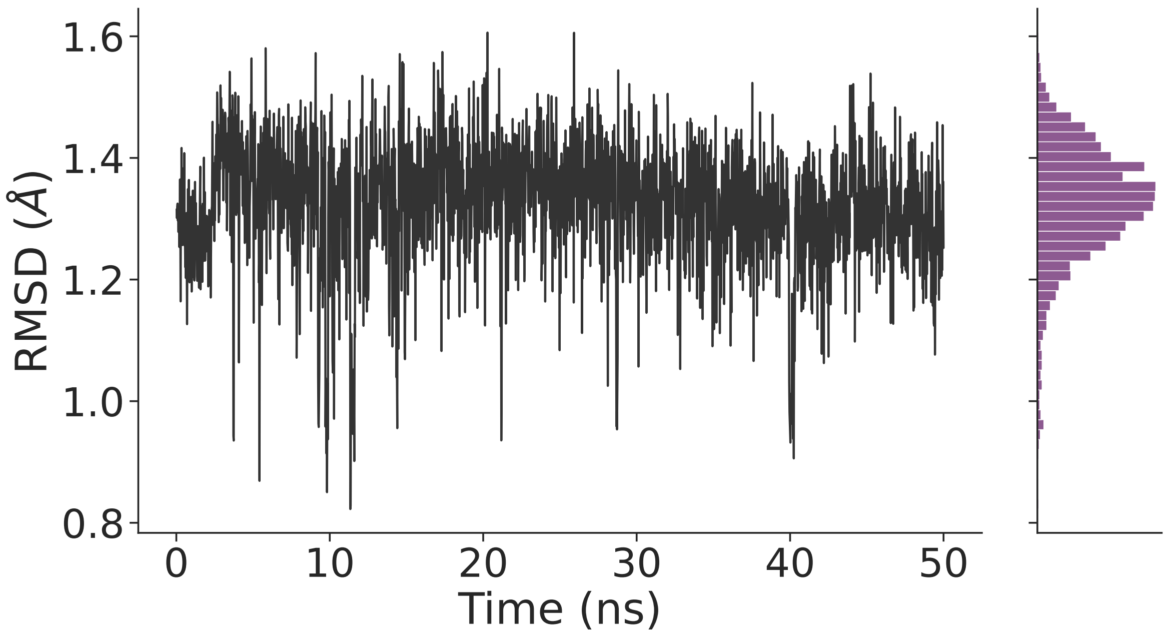

Figure S8: Active site RMSD from 50 ns MD simulation of *ligand 16* bound to VIM-2 simulated with the restraint approach.

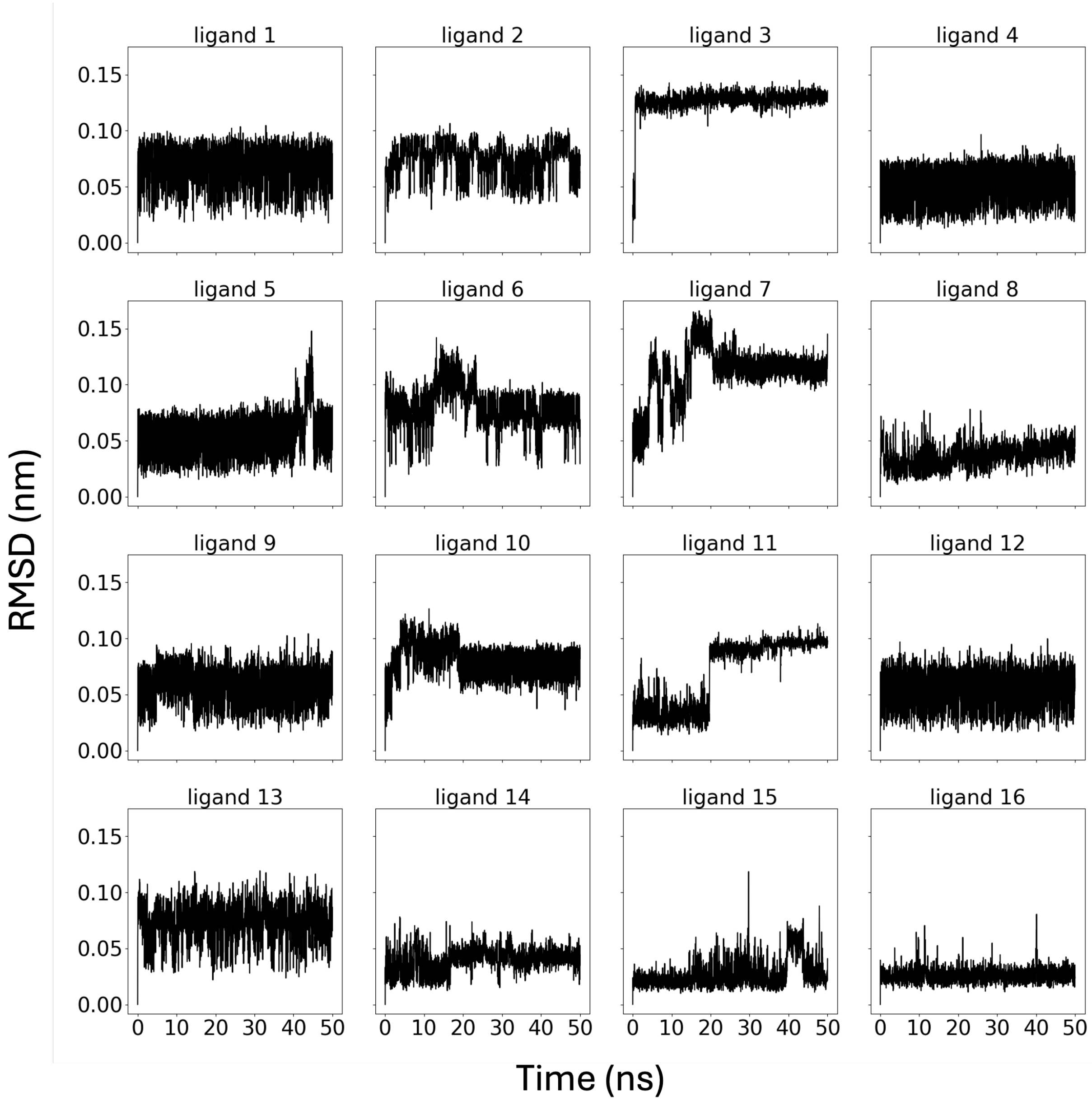

Figure S9: RMSDs computed from 50 ns MD simulations ran with the restraint approach for each ligand bound to VIM-2.

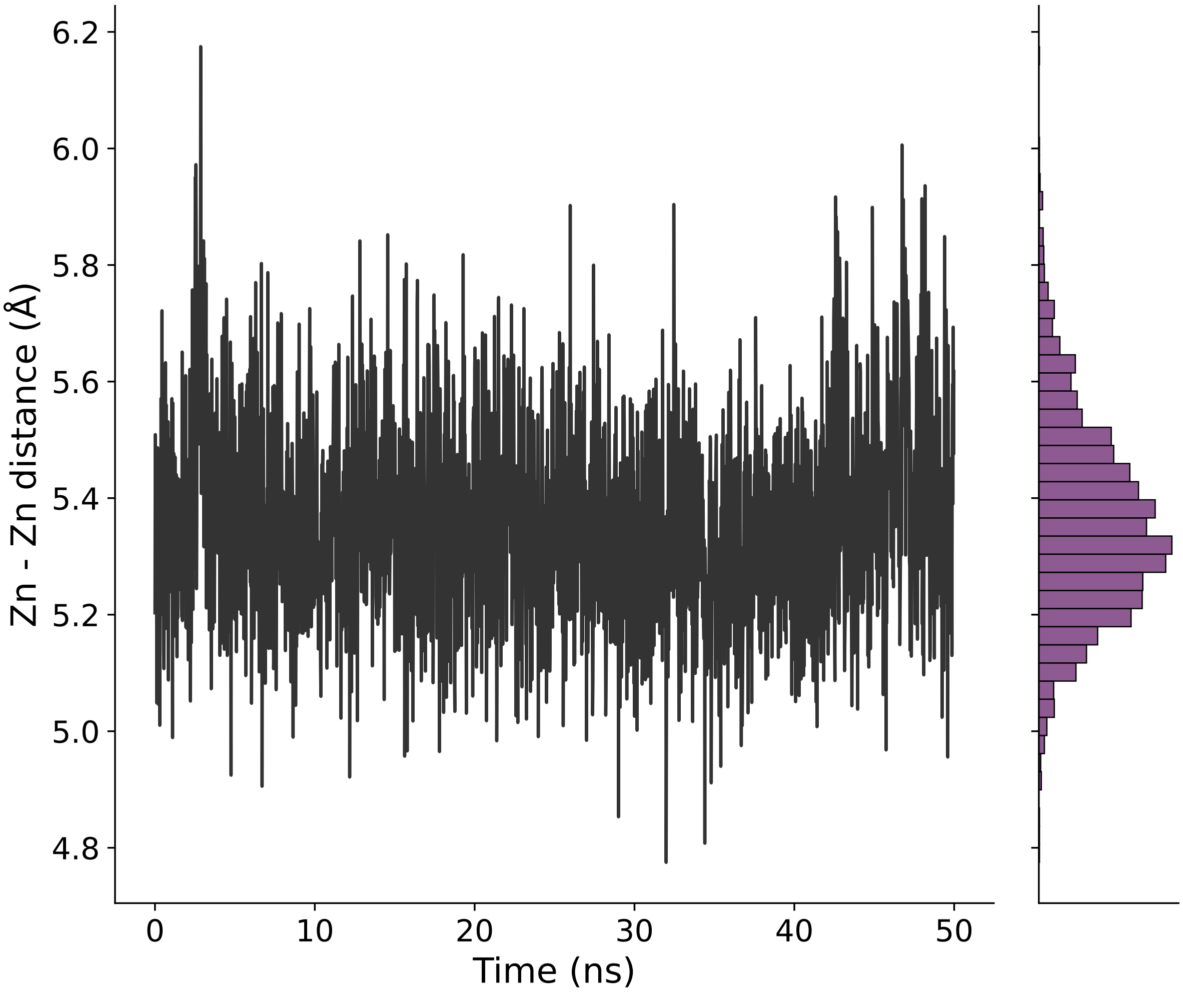

Figure S10: Zinc–zinc distance from 50 ns MD simulation of VIM-2 with *ligand 16* bound and using the restraint approach.

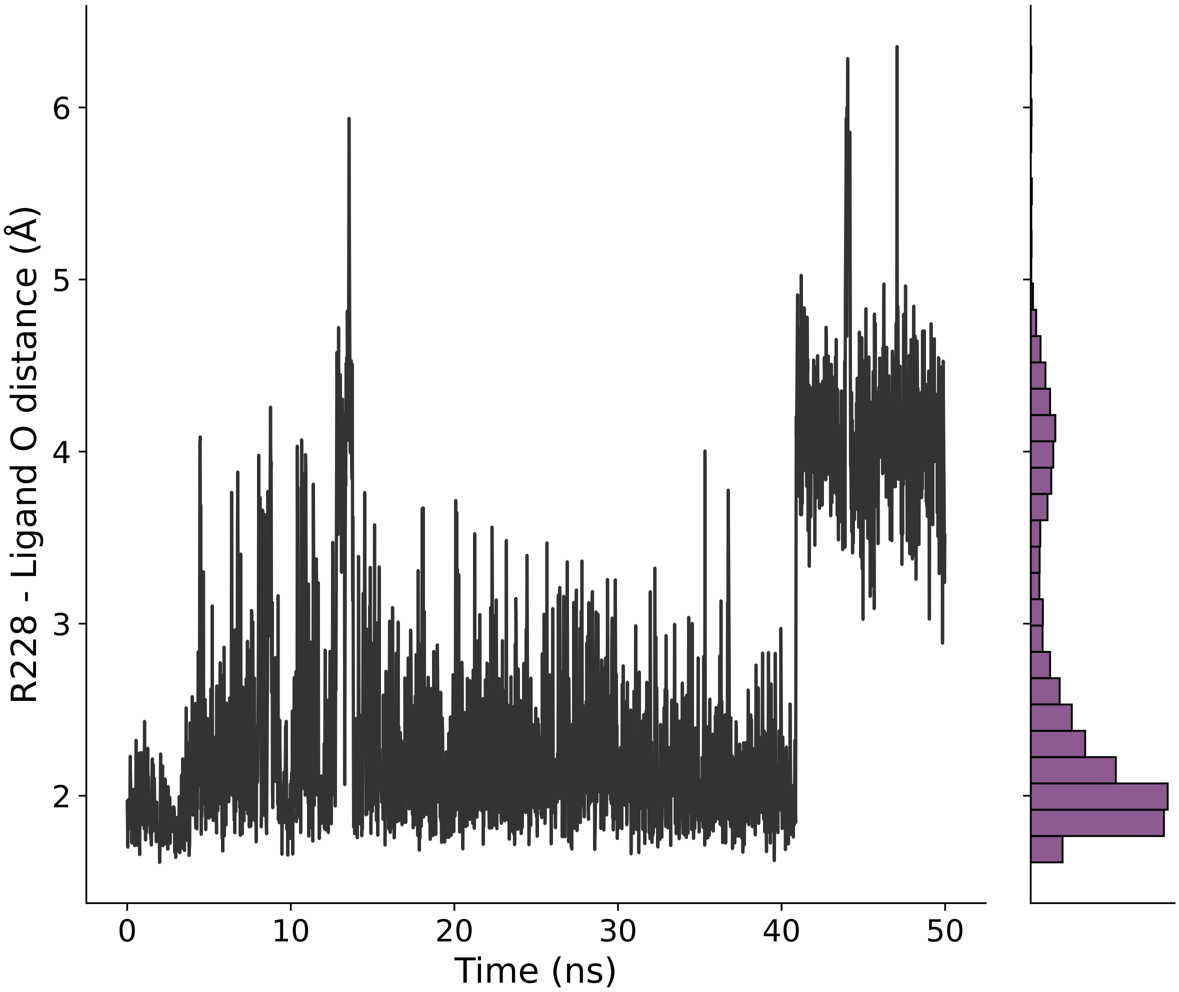

Figure S11: *Ligand 16* to R288 distance from 50 ns MD simulation of VIM-2 with *ligand 16* bound and using the restraint approach.

The UAFF approach

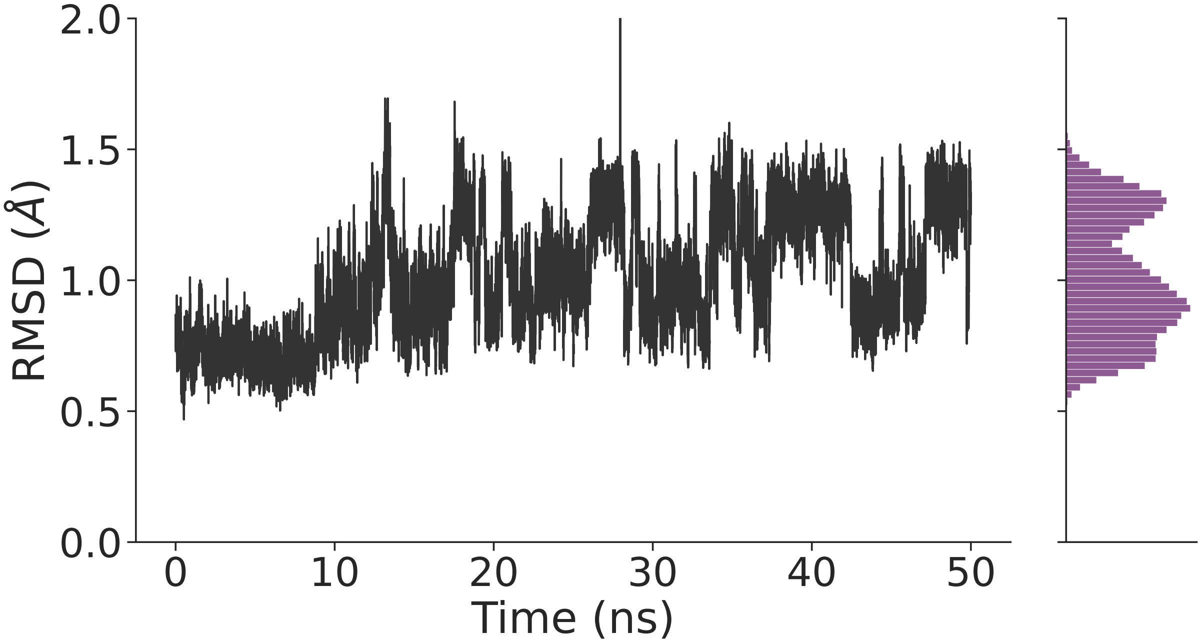

Figure S12: Active site RMSD from 50 ns MD simulation of *ligand 16* bound to VIM-2 simulated with the UAFF method.

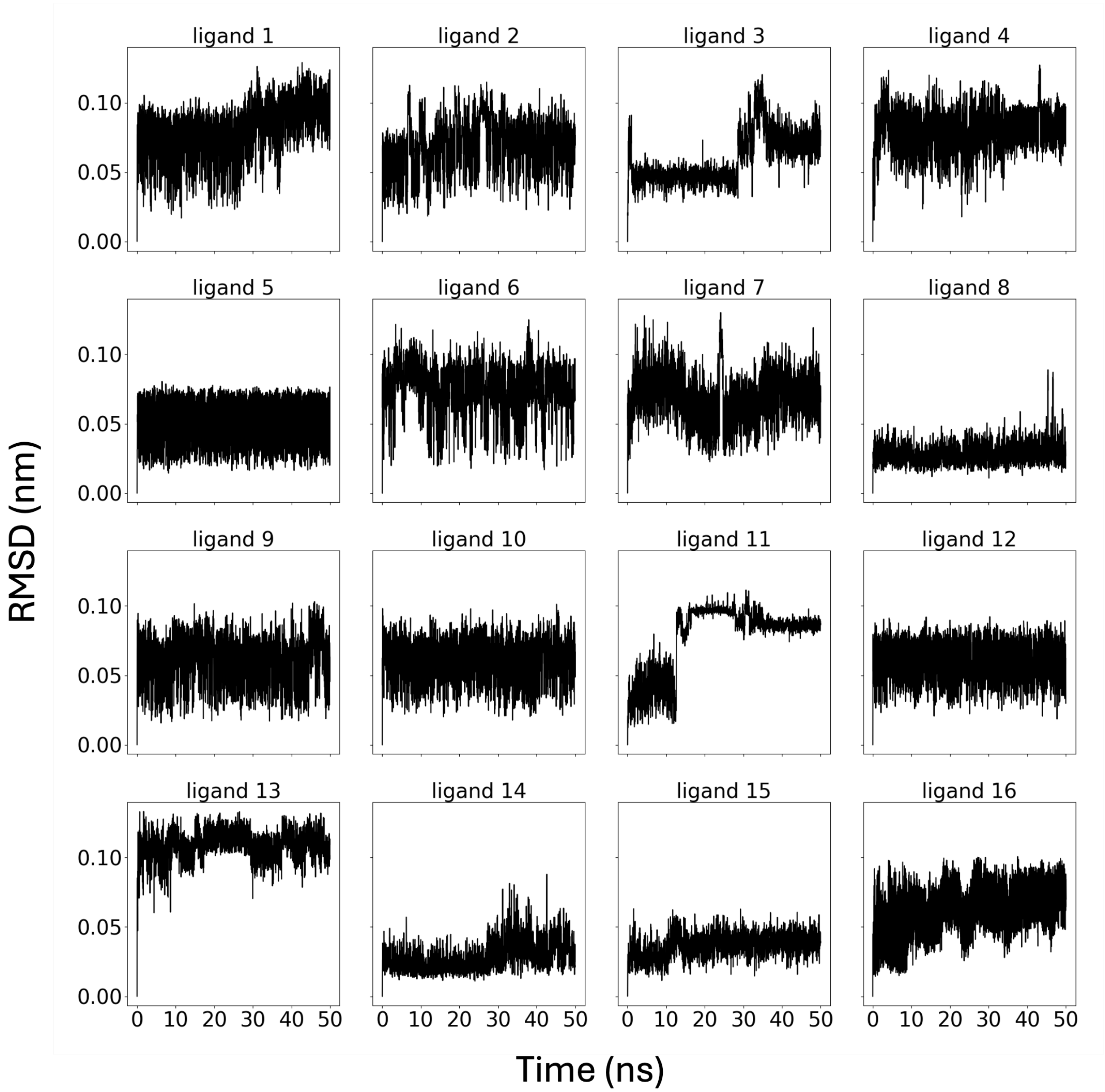

Figure S13: RMSDs computed from 50 ns MD simulations run with the UAFF model for each ligand bound to VIM-2.

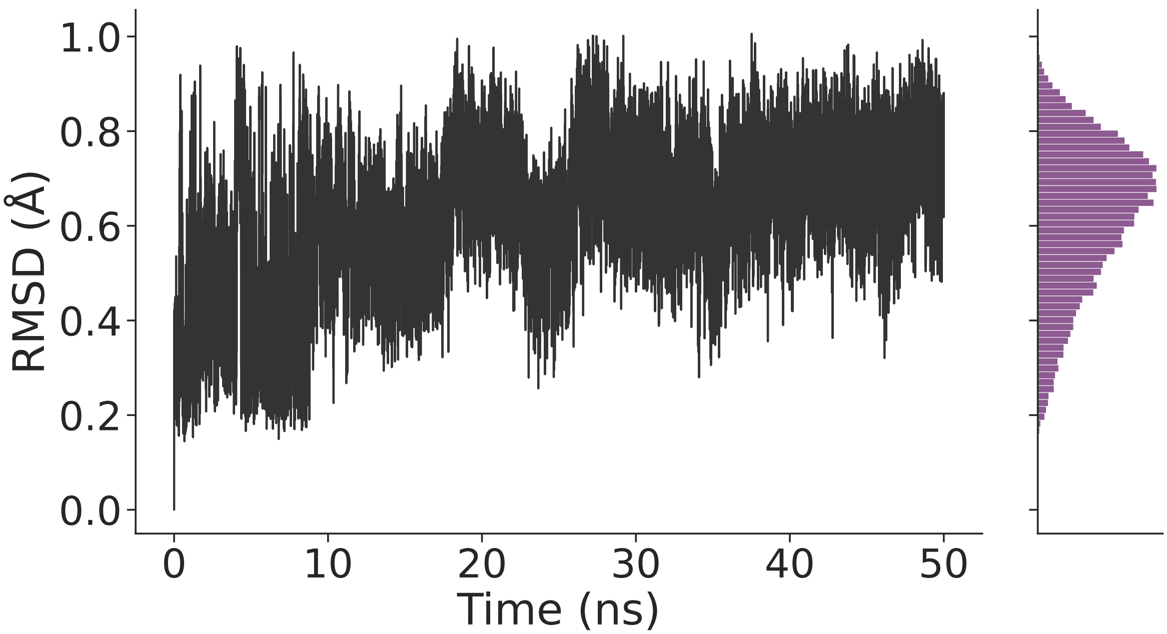

Figure S14: *Ligand 16* RMSD from 50 ns MD simulation using the UAFF method.

RBFE plots

LOMAP network

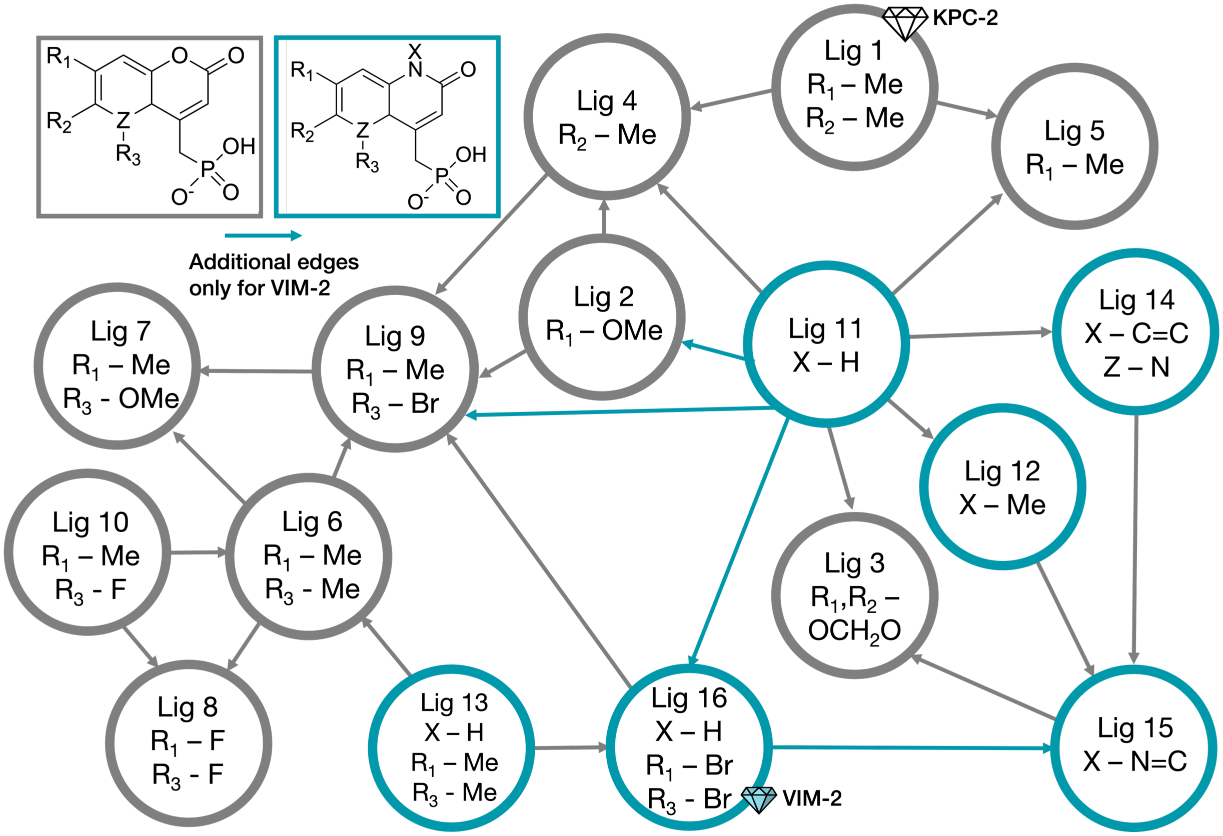

Figure S15: LOMAP generated perturbation network for our RBFE simulations. The circles are color-coordinated based on the scaffold being a coumarin (gray) or dihydroquinolinone (teal). The dimond symbols indicate the crystal structure pose for KPC-2 and VIM-2. The arrows colored in teal were only run for VIM-2 RBFE simulations, as there were fewer ligands tested experimentally for VIM-2 than for KPC-2.

KPC-2

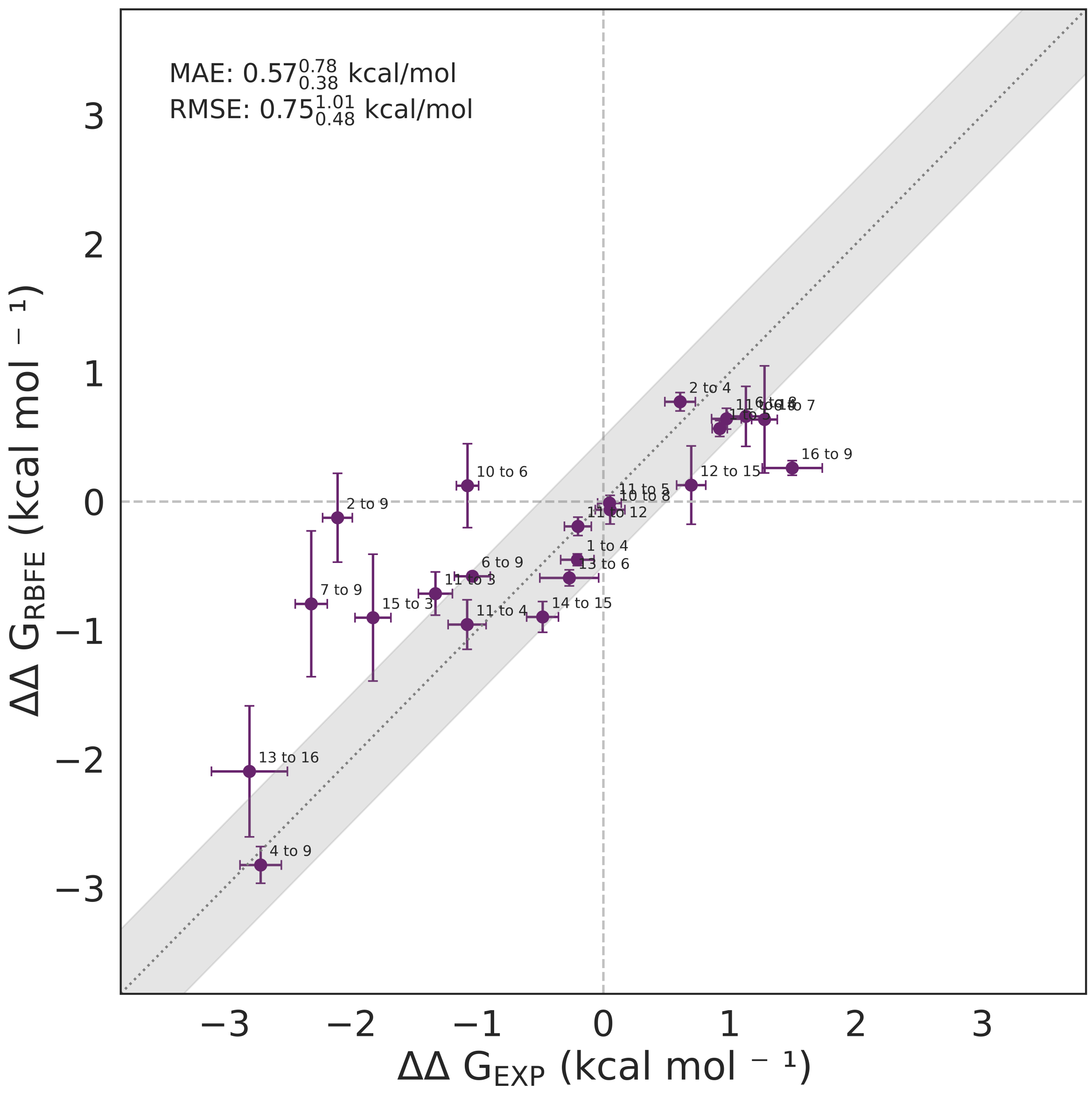

Figure S16: $\Delta\Delta G$ correlation to experiment for KPC-2.

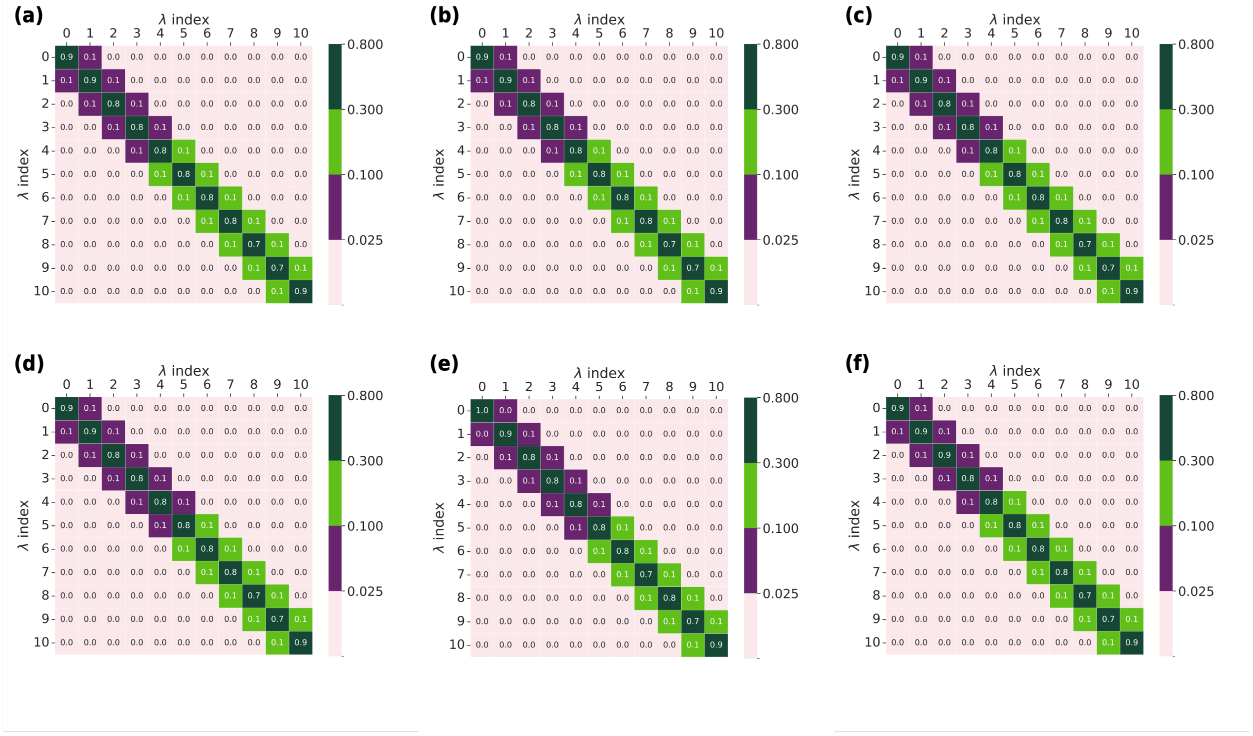

Figure S17: Overlap matrix of transforming *ligand 2* to *ligand 9* for the unbound (a-c) and bound stages (d-f) for each of the repeats from the KPC-2 simulations.

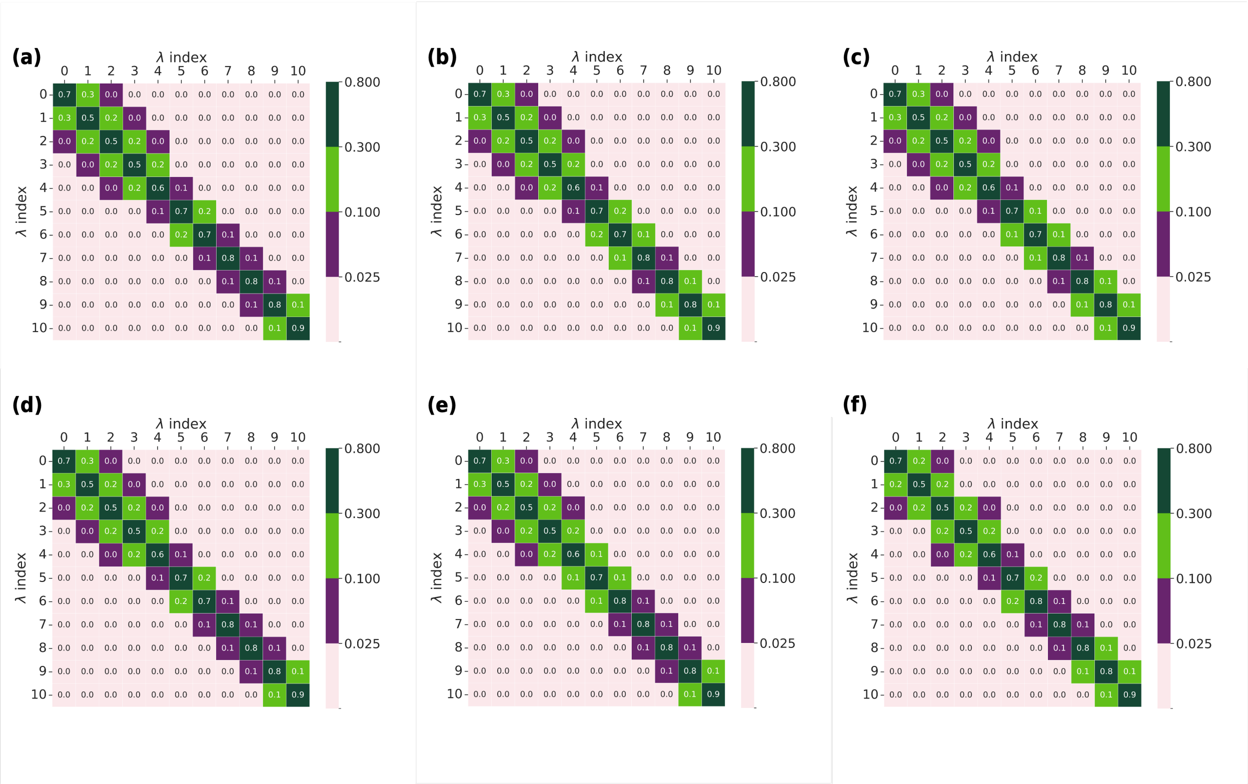

Figure S18: Overlap matrix of transforming *ligand 15* to *ligand 3* for the unbound (a-c) and bound stages (d-f) for each of the repeats from the KPC-2 simulations.

VIM-2

The restraint approach

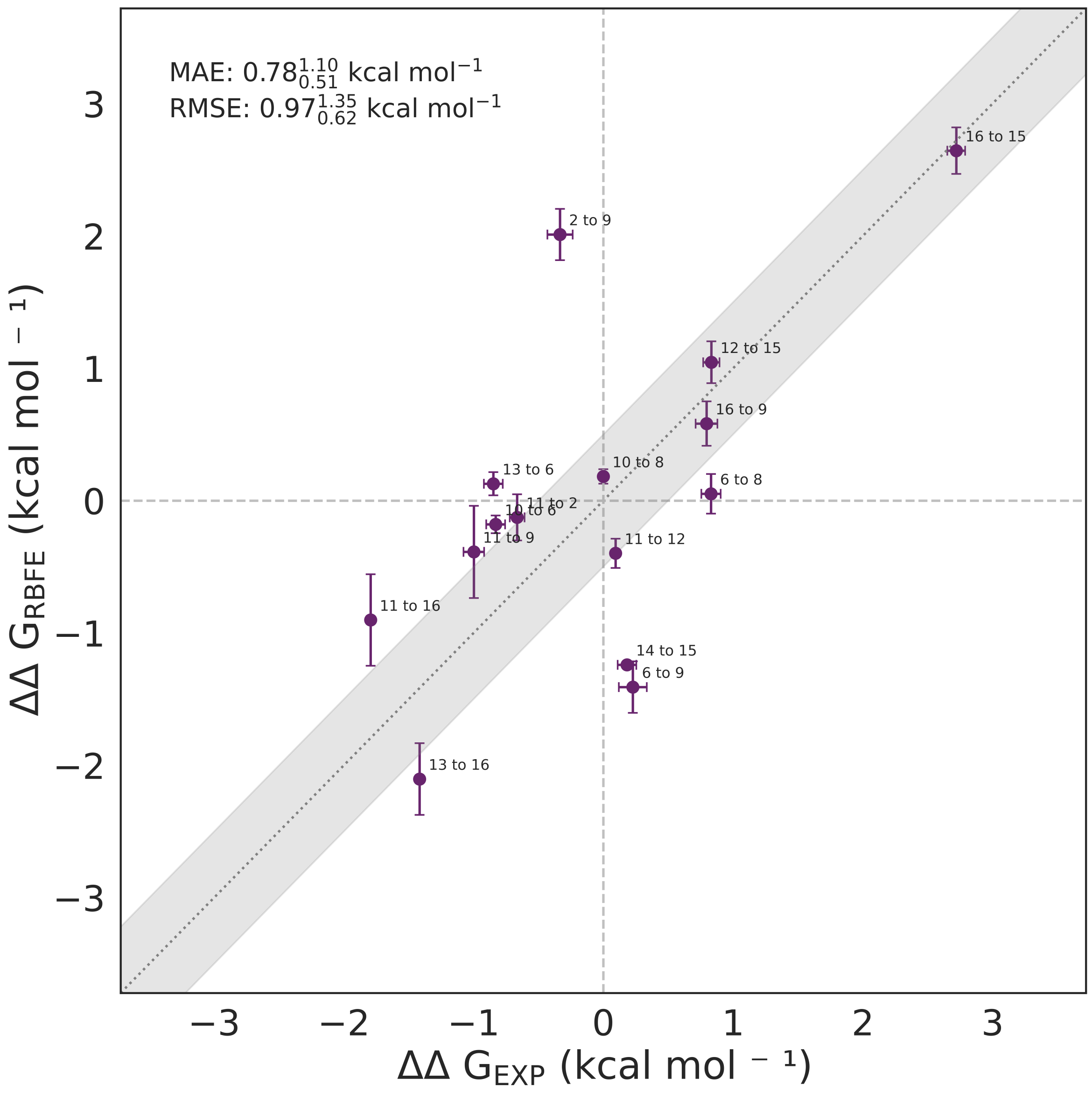

Figure S19: $\Delta\Delta G$ correlation to experiment for VIM-2 with the restraint approach.

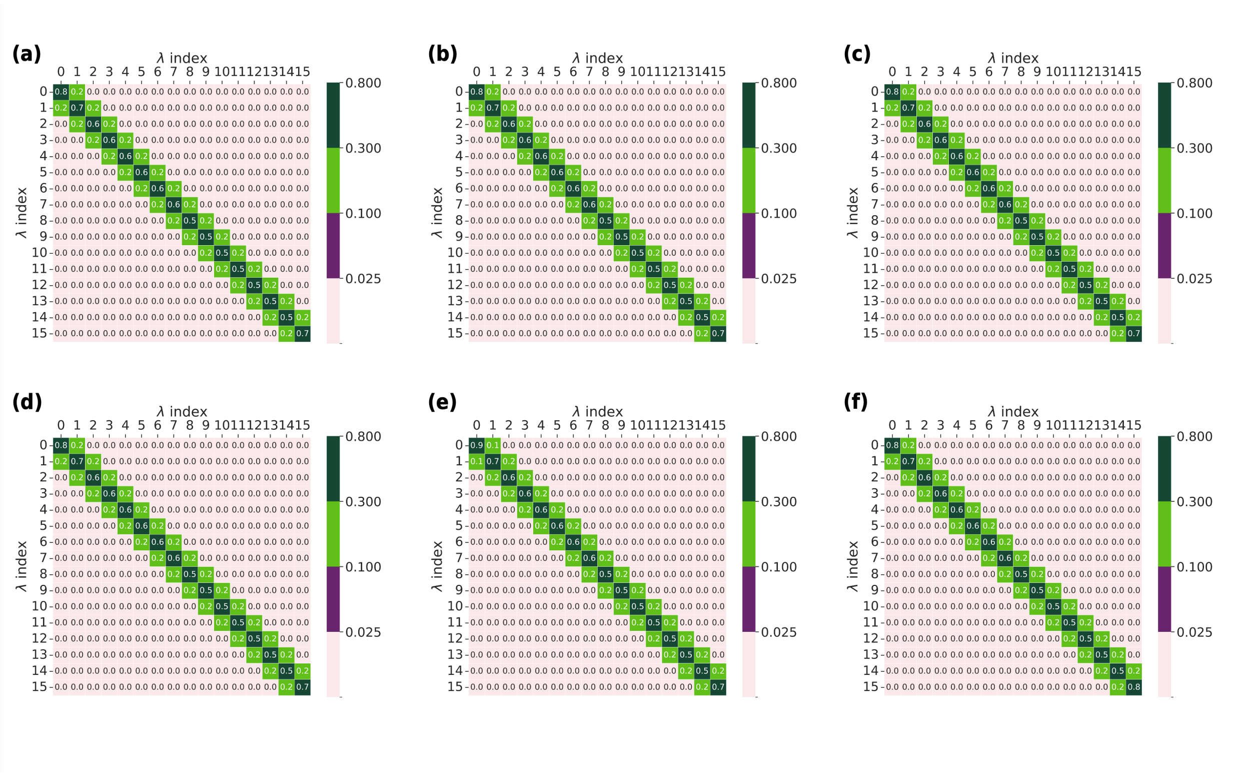

Figure S20: Overlap matrix of transforming *ligand 2* to *ligand 9* in the VIM-2 restrained approach for the unbound (a-c) and bound stages (d-f) for each of the repeats.

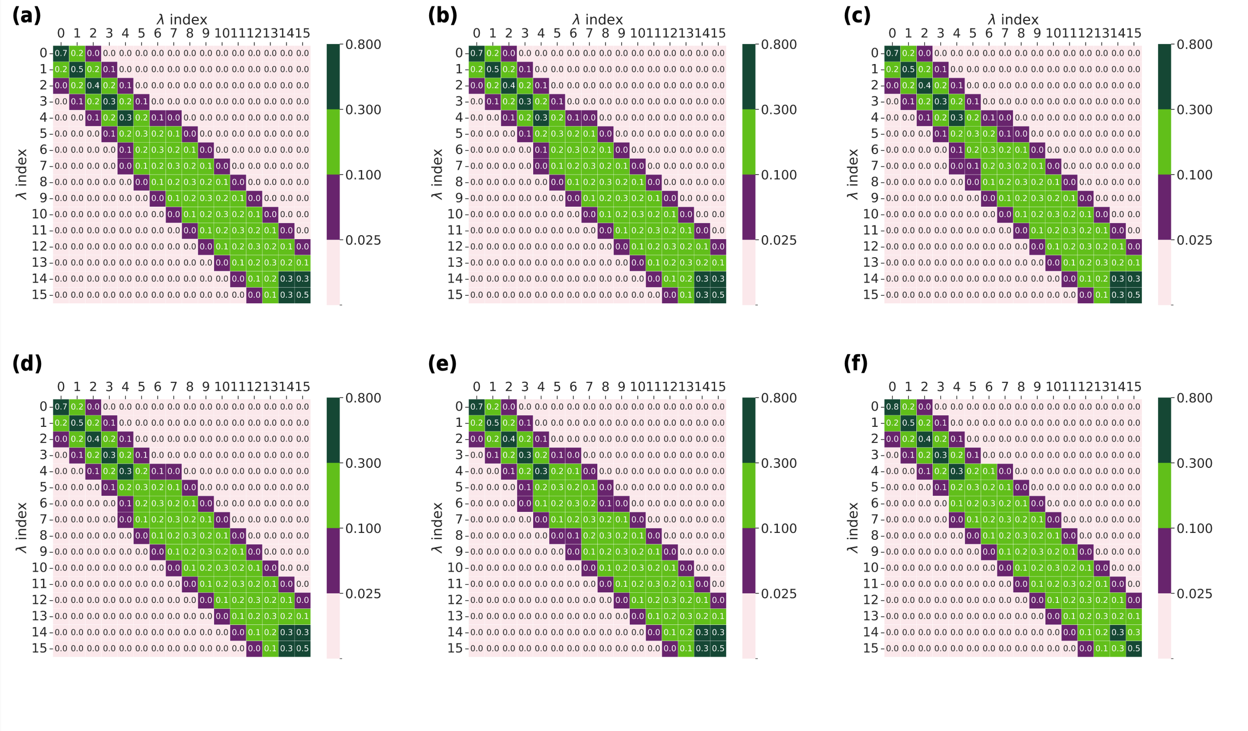

Figure S21: Overlap matrix of transforming *ligand 13* to *ligand 16* in the VIM-2 restrained approach for the unbound (a-c) and bound stages (d-f) for each of the repeats.

The Upgraded Amber Force Field method

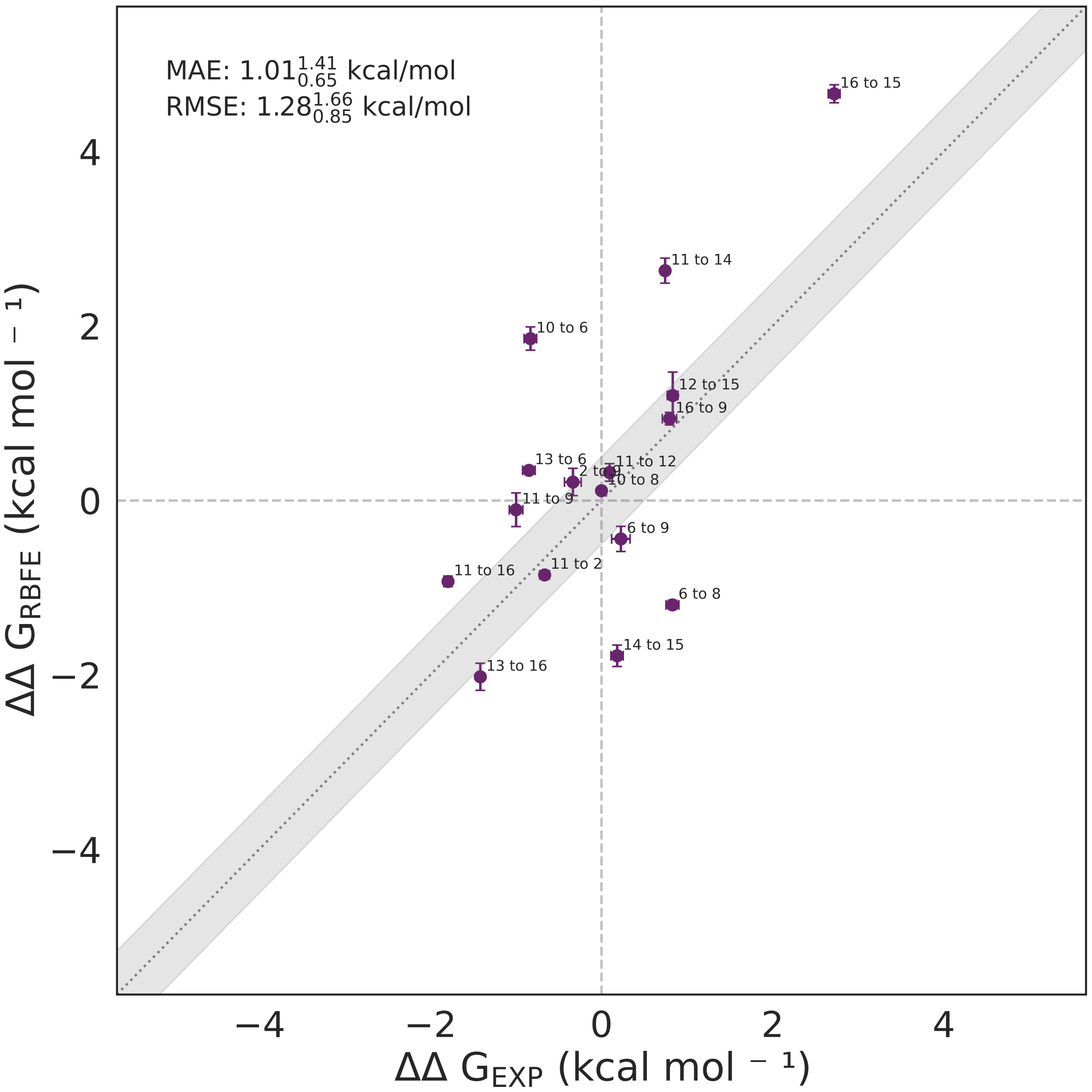

Figure S22: $\Delta\Delta G$ correlation to experiment for VIM-2 with the UAFF method.

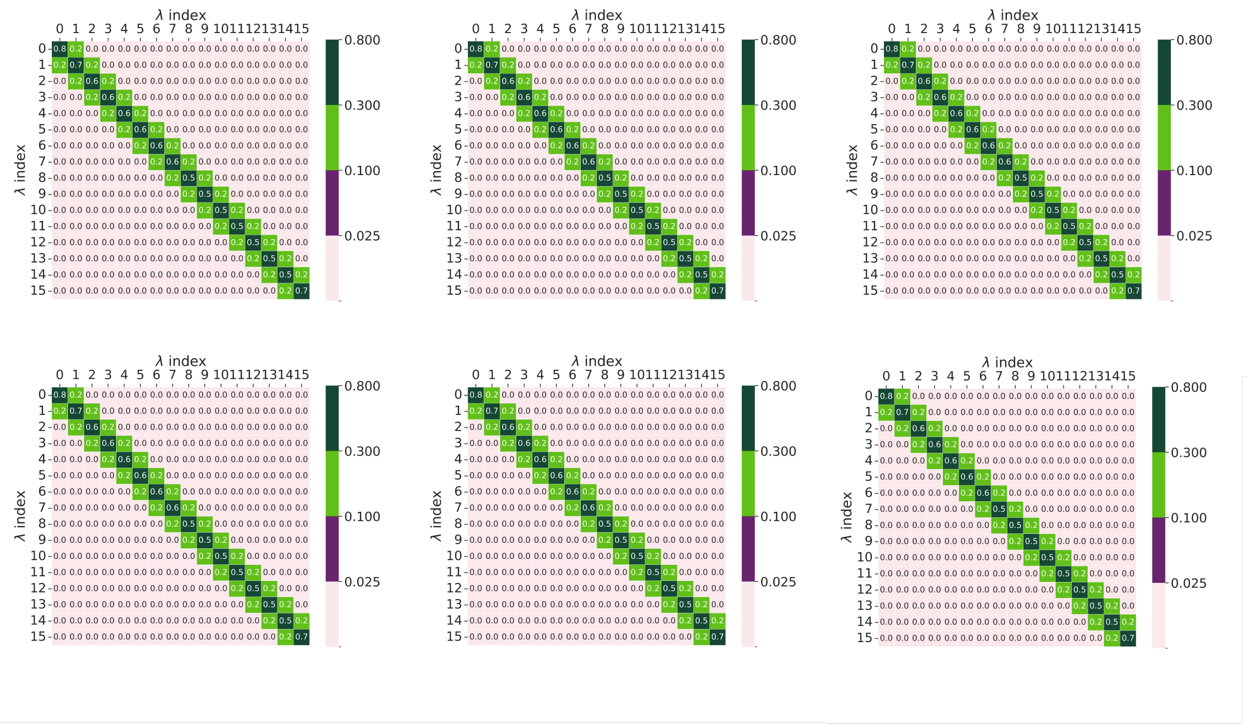

Figure S23: Overlap matrix of transforming *ligand 2* to *ligand 9* in the VIM-2 UAFF method for the unbound (a-c) and bound stages (d-f) for each of the repeats.

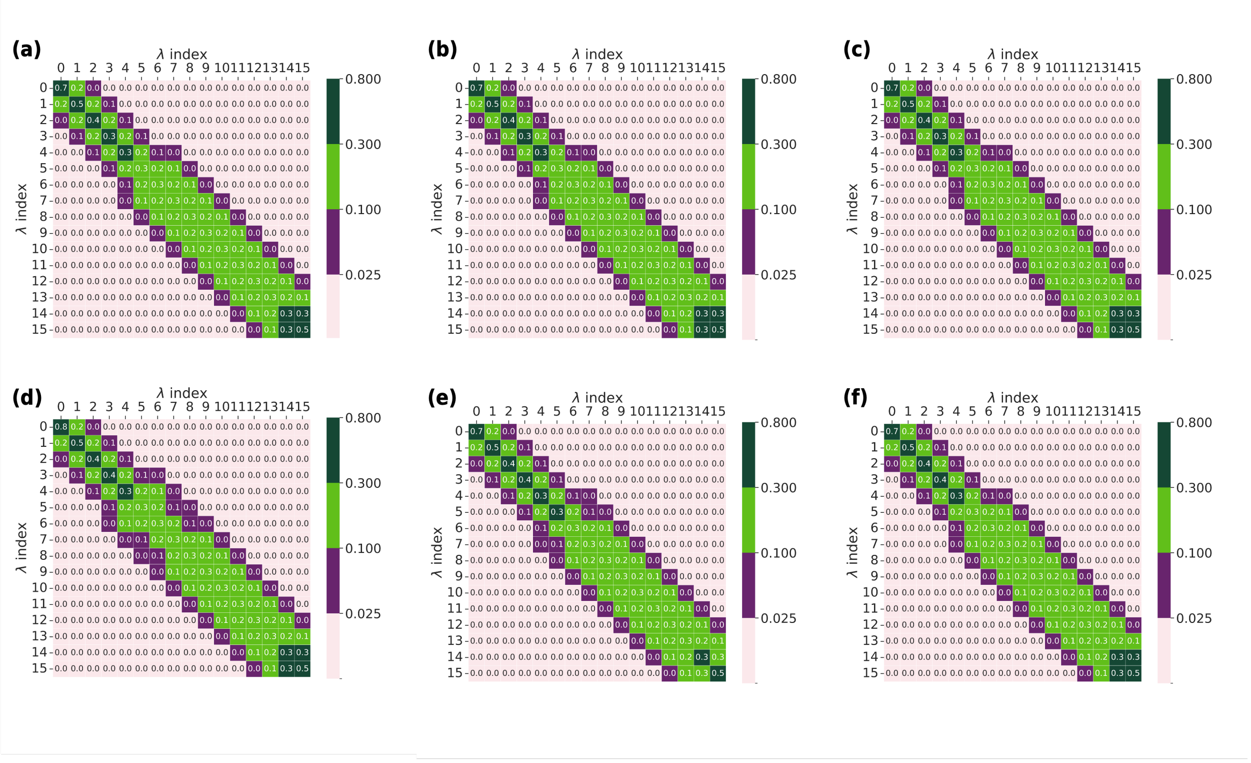

Figure S24: Overlap matrix of transforming *ligand 13* to *ligand 16* in the VIM-2 UAFF method for the unbound (a-c) and bound stages (d-f) for each of the repeats.

Statistical analysis

Table S2: Mann Whitney U statistic and their corresponding p-values calculated between the UAFF and restraint models. All p-values are below $\alpha = 0.05$, suggesting that the differences in these statistics between the two models are statistically significant.

**Statistic**	**Mann Whitney U statistic**	**p-value**
Pearson R	$1.5{\cdot}10^5$	$5{,}6{\cdot}10^{-158}$
Spearman ρ	$2.5{\cdot}10^5$	$7.0{\cdot}10^{-81}$
MAE	$1.8{\cdot}10^5$	$1.1{\cdot}10^{-132}$
RMSE	$2.5{\cdot}10^5$	$9.4{\cdot}10^{-87}$

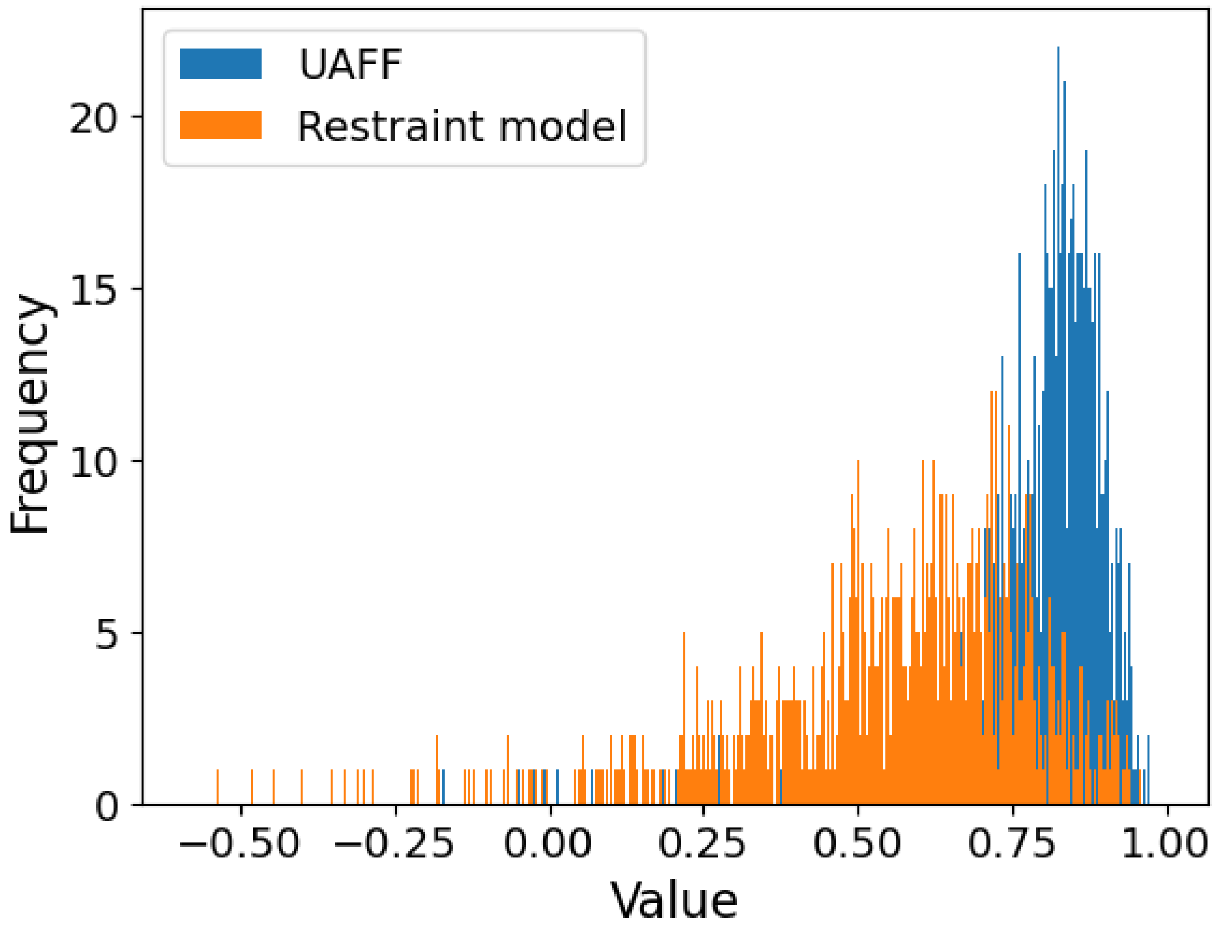

Figure S25: Bootstrap distributions of the Pearson R correlation coefficient calculated between experimental values and the UAFF model (blue) and the restraint model (orange).

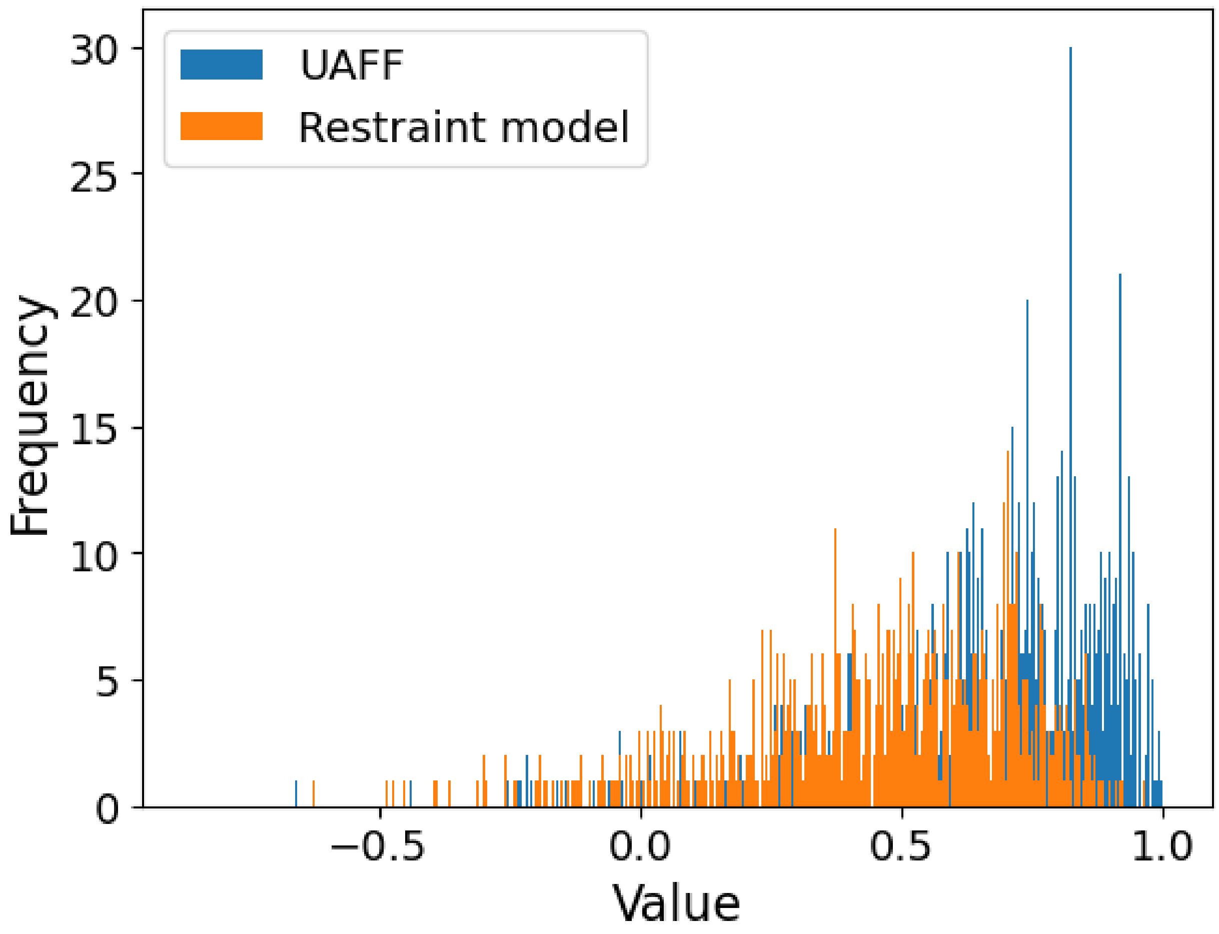

Figure S26: Bootstrap distributions of the Spearman ρ rank correlation coefficient calculated between experimental values and the UAFF model (blue) and the restraint model (orange).

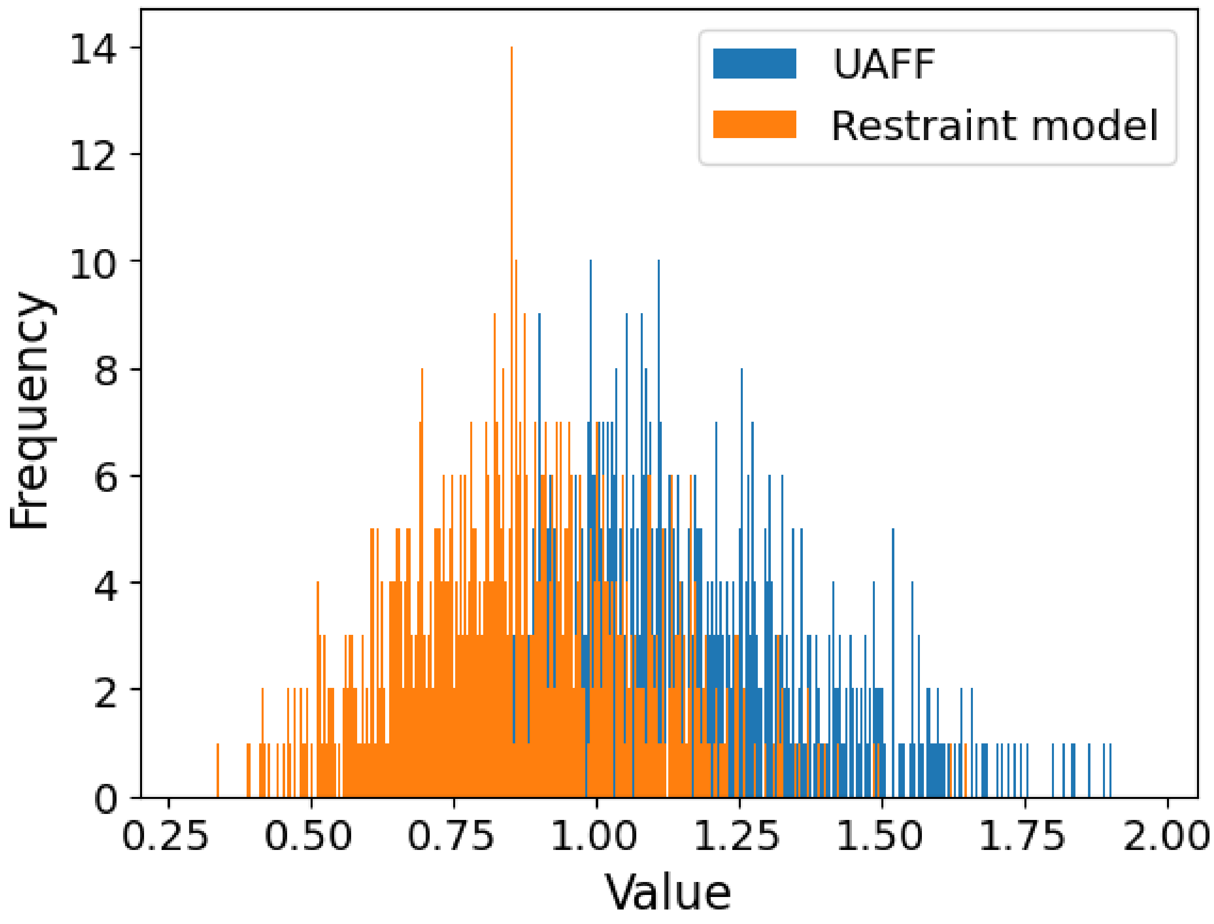

Figure S27: Bootstrap distributions of the MAE calculated between experimental values and the UAFF model (blue) and the restraint model (orange).

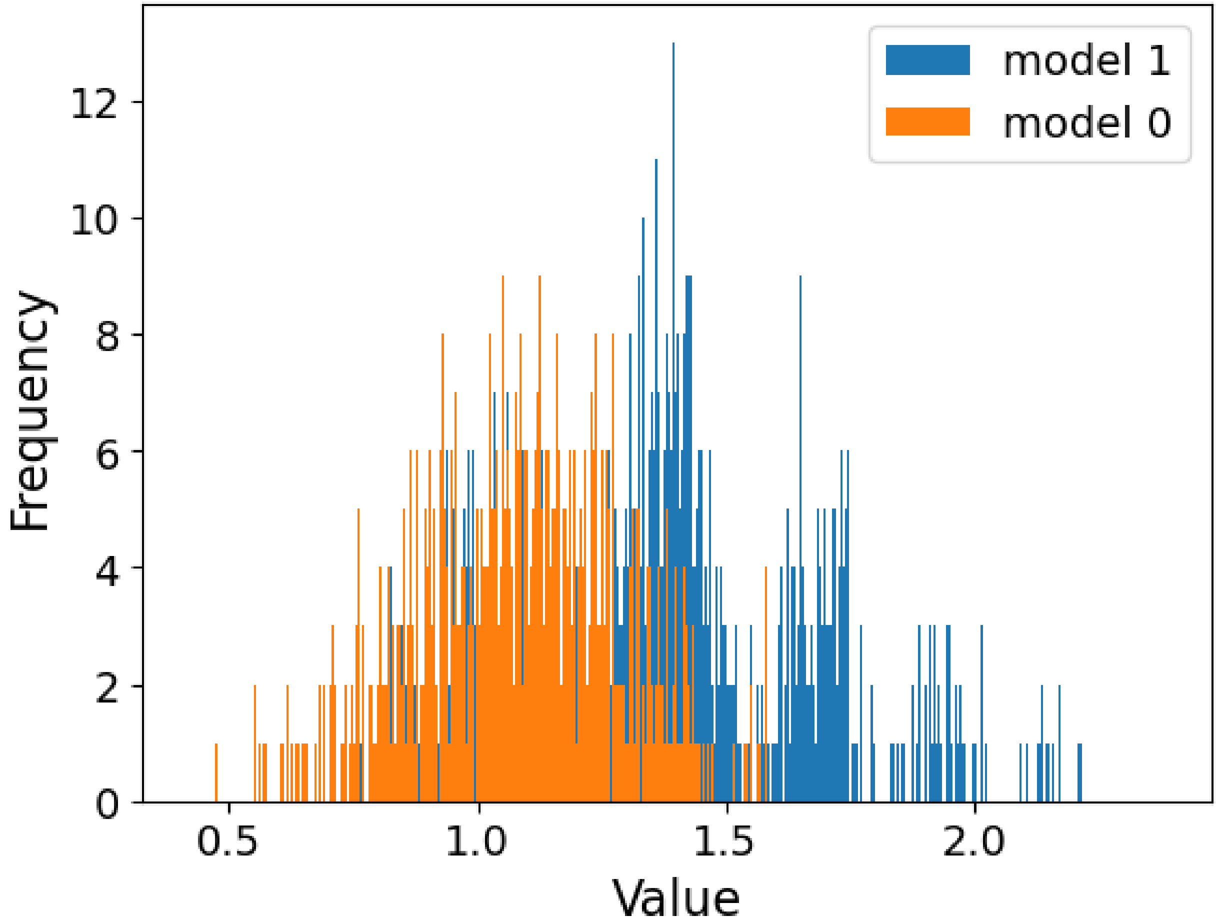

Figure S28: Bootstrap distributions of the RMSE calculated between experimental values and the UAFF model (blue) and the restraint model (orange).

References

(1) Pemberton, O. A.; Jaishankar, P.; Akhtar, A.; Adams, J. L.; Shaw, L. N.; Renslo, A. R.; Chen, Y. Heteroaryl Phosphonates as Noncovalent Inhibitors of Both Serine- and Metallocarbapenemases. *J. Med. Chem.* **2019**, *62*, 8480–8496.
